# Genome-wide analysis of SARS-CoV-2 virus strains circulating worldwide implicates heterogeneity

**DOI:** 10.1038/s41598-020-70812-6

**Published:** 2020-08-19

**Authors:** M. Rafiul Islam, M. Nazmul Hoque, M. Shaminur Rahman, A. S. M. Rubayet Ul Alam, Masuda Akther, J. Akter Puspo, Salma Akter, Munawar Sultana, Keith A. Crandall, M. Anwar Hossain

**Affiliations:** 1 Department of Microbiology, University of Dhaka, Dhaka, 1000 Bangladesh; 2 Department of Gynecology, Obstetrics and Reproductive Health, Bangabandhu Sheikh Mujibur Rahman Agricultural University, Gazipur, 1706 Bangladesh; 3 Department of Microbiology, Jashore University of Science and Technology, Jashore, 7408 Bangladesh; 4 Department of Microbiology, Jahangirnagar University, Savar, Dhaka, 1342 Bangladesh; 5 Computational Biology Institute, Milken Institute School of Public Health, The George Washington University, Washington, USA; 6 Present Address: Jashore University of Science and Technology, Jashore, 7408 Bangladesh

**Keywords:** Computational biology and bioinformatics, Evolution, Genetics, Microbiology

## Abstract

Severe acute respiratory syndrome coronavirus-2 (SARS-CoV-2), a novel evolutionary divergent RNA virus, is responsible for the present devastating COVID-19 pandemic. To explore the genomic signatures, we comprehensively analyzed 2,492 complete and/or near-complete genome sequences of SARS-CoV-2 strains reported from across the globe to the GISAID database up to 30 March 2020. Genome-wide annotations revealed 1,516 nucleotide-level variations at different positions throughout the entire genome of SARS-CoV-2. Moreover, nucleotide (nt) deletion analysis found twelve deletion sites throughout the genome other than previously reported deletions at coding sequence of the ORF8 (open reading frame), spike, and ORF7a proteins, specifically in polyprotein ORF1ab (n = 9), ORF10 (n = 1), and 3´-UTR (n = 2). Evidence from the systematic gene-level mutational and protein profile analyses revealed a large number of amino acid (aa) substitutions (n = 744), demonstrating the viral proteins heterogeneous. Notably, residues of receptor-binding domain (RBD) showing crucial interactions with angiotensin-converting enzyme 2 (ACE2) and cross-reacting neutralizing antibody were found to be conserved among the analyzed virus strains, except for replacement of lysine with arginine at 378th position of the cryptic epitope of a Shanghai isolate, hCoV-19/Shanghai/SH0007/2020 (EPI_ISL_416320). Furthermore, our results of the preliminary epidemiological data on SARS-CoV-2 infections revealed that frequency of aa mutations were relatively higher in the SARS-CoV-2 genome sequences of Europe (43.07%) followed by Asia (38.09%), and North America (29.64%) while case fatality rates remained higher in the European temperate countries, such as Italy, Spain, Netherlands, France, England and Belgium. Thus, the present method of genome annotation employed at this early pandemic stage could be a promising tool for monitoring and tracking the continuously evolving pandemic situation, the associated genetic variants, and their implications for the development of effective control and prophylaxis strategies.

## Introduction

Severe acute respiratory syndrome (SARS) is an emerging pneumonia-like respiratory disease of human, which was reported to be re-emerged in Wuhan city of China in December 2019^1^. The identified causative agent is found to be a highly contagious novel beta-coronavirus 2 (SARS-CoV-2). Similar to other known SARS-CoV and SARS-related coronaviruses (SARSr-CoVs)^2,3^, the viral RNA genome of SARS-CoV-2 encodes several smaller open reading frames (ORFs) such as ORF1ab, ORF3a, ORF6, ORF7a, ORF7b, ORF8 and ORF10 located in the 3′ region. These ORFs are predicted to encode for the replicase polyprotein, the spike (S) glycoprotein, envelope (E), membrane (M), nucleocapsid (N) proteins, accessory proteins, and other non-structural proteins (NSP)^3–5^.

However, the ongoing rapid transmission and global spread of SARS-CoV-2 have raised critical questions about the evolution and adaptation of the viral population driven by mutations, deletions and/or recombination as it spreads across the world encountering diverse host immune systems and various counter-measures^6^. Initial phylogenomic analysis of three super-clades (S, V, and G) isolated from the outbreaks of distinct geographic locations (China, USA and Europe) within SARS-CoV-2 showed little evidence of local/regional adaptation, suggesting instead that viral evolution is mainly driven by genetic drift and founder events^7^. Nevertheless, several reports predict possible adaptation at the nt, amino acid (aa), and structural heterogeneity in the viral proteins, especially in the S protein^8,9^. Interestingly, Shen et al. reported even intra-host viral evolution among the patients after infection, which might be related to its virulence, transmissibility, and/or evolution due to immune response^10^. However, the previous reports have the limitations of considering a very few representative complete genomes covering only a few countries, targeting clade/group based consensus sequence, comparison to the Wuhan Refseq genome, and focusing on the structural proteins.

The mutation and evolution rate of RNA viruses is dramatically high, up to a million times higher than that of their hosts, and this high rate is correlated with virulence modulation and evolvability, traits considered beneficial for viral adaptation^11^. The genomes of the RNA viruses can accrue genetic differences while being spatially disseminated during an individual outbreak^12^. Deletions within the viral genome is a natural phenomenon, almost inevitably related to the attenuation of virus, however it can sometimes be linked with more severe infection^13–15^. In case of SARS-CoV-2, several reports pointed out the deletions in throughout the viral genome, often producing deletion variants of non-structural and accessory proteins that may have direct implication upon viral infectivity ^5,16–19^. However, still there is a lack of studies bridging all the deletions taken part in the entire genome of SARS-CoV-2 globally, which may contribute to understand the pathogenic dynamics of the virus over time.

The genetic differences among SARS-CoV-2 strains sampled from diverse locations could therefore be linked with their geographical distributions. This rapidly evolving virus is capable of adapting swiftly to the diverge environments. The rapid spread of the COVID-19 pandemic is a sparking challenge for the entire world, and climate change may also affect the emergence of this novel coronavirus. Exactly how is highly uncertain but it is worth keeping in mind that most novel diseases originate in wildlife before they spread to humans^20^. The recent emerging evidences suggested that the transmission of COVID-19 is associated with weather conditions, with dry and cold conditions appeared to boost the spreading of the infections^21,22^. Moreover, the per day mortality rate of COVID-19 is positively associated with diurnal temperature range (DTR) but negatively with relative humidity^21^. The integration of geographical and climatic data with genetic mutation analysis promises to provide a fuller understanding of the origins, dispersal and dynamics of the evolving SARS-CoV-2 virus. Therefore, this study has targeted the genome-wide mutational spectra covering nucleotide, aa and deletion mutations in 2,492 complete (and/or near complete) genomes of SARS-CoV-2 retrieved from the global initiative on sharing all influenza data (GISAID) (https://www.gisaid.org/) up to 30 March 2020 (Supplementary Fig. 1, Supplementary Table 1a). Furthermore, these preliminary data of SARS-CoV-2 genome sequences belonged to different geographic and climatic conditions for inference on the evolution of this novel virus.

**Figure 1:**
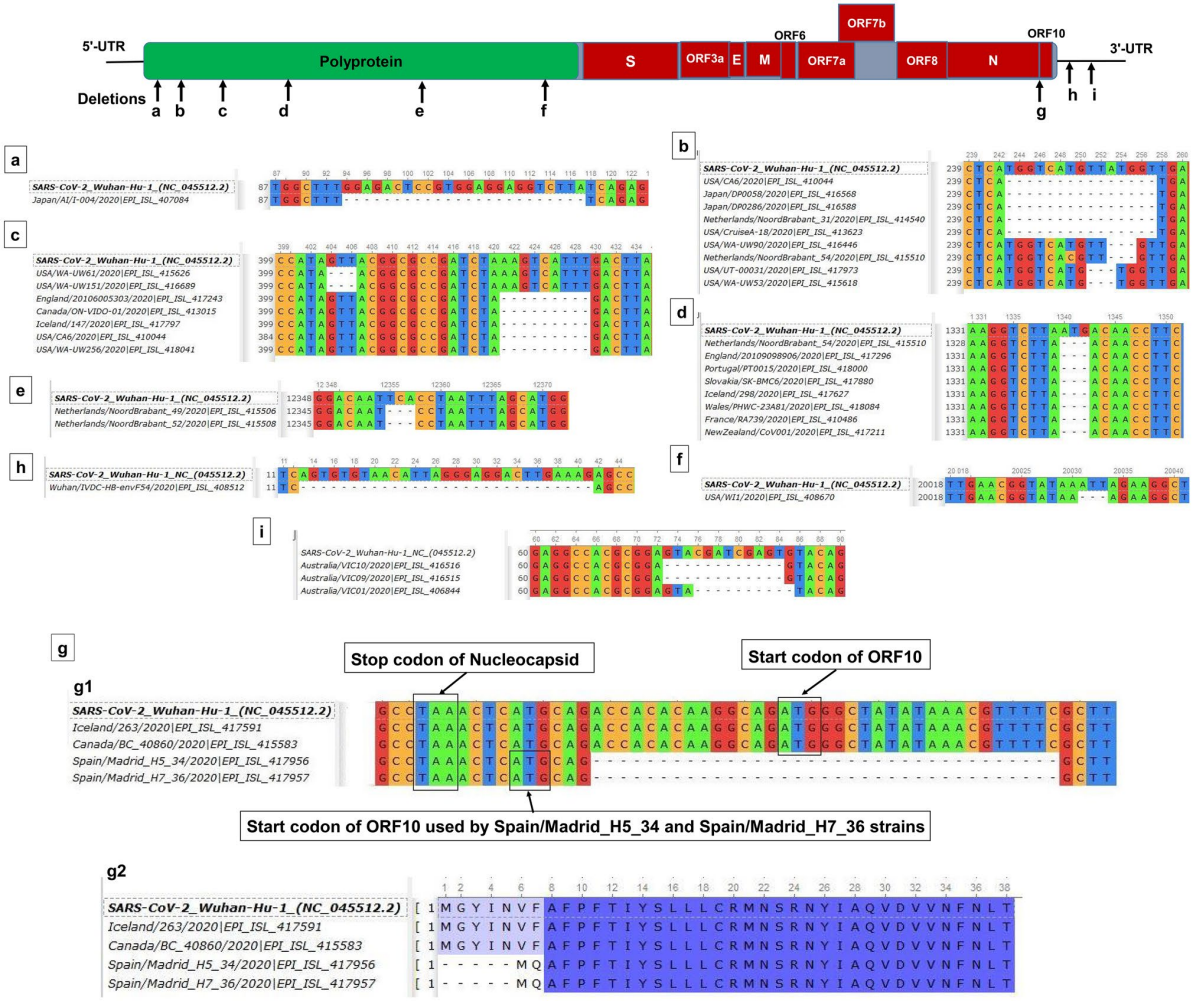
Genomic deletion analysis of SARS-CoV-2. Genomic deletion analysis of SARS-CoV-2 strains identified (**a**) 24 (nt) deletions in NSP1 in a Japanese strain; (**b**) 15-nt deletions in NSP1 of viral strains from USA, Japan and the Netherlands alongside three-nt deletions in USA and Netherlands; (**c**) three-nt deletion in NSP1 of American strains and very adjacent to that, nine-nucleotide deletion of strains from the USA, England and Canada and Iceland; (**d**) three-nucleotide deletions in NSP2 were observed in 99 strains from Netherlands, England, Portugal, Slovakia, Iceland, Wales, France and New Zealand (representatives from each countries were shown); (**e**) NSP8 undergoes three-nt deletion in Netherlandian/dutch/hollanders strains; (**f**) three-nucleotide deletion in NSP15 of USA strain; (**g**-**g1**) 35nt deletion, including start codon position of ORF10 of Spain strains, and the start codon in spacer position, has been used for ORF10 coding; and as a result, (**g**-**g2**) five aa residues deletion in those strains starting from position 1 to 5. Deletion of (**h**) 29-nt reported from Wuhan, and (**i**) 10-nt in 3′-UTR of strains belonged to Australia. The position of nt represents the starting position from each ORFs, for instance, position of ORF1ab was considered for the NSPs. MAFFT online tool^36^ was used for alignment, and Unipro-UGENE^40^ used for visualization.

## Results and discussions

### Mutations throughout the SARS-CoV-2 genomes: a special focus on the RBD of spike protein

Nucleotide (nt) sequence alignment revealed a total of 1,516 mutations (synonymous vs nonsynonymous ratio = 2.5:1) across the entire set of genomes of the SARS-CoV-2 strains compared to the NCBI reference strain, Wuhan-Hu-1 (Accession NC_045512). Of the identified nt substitutions, 661 were found in polyprotein regions of SARS-CoV-2 while the structural proteins regions had 382nt mutations comprising 183, 34, 17 and 148 mutations in S, M, E and N proteins, respectively. In addition, the ORF3a, ORF6, ORF7ab, ORF8, and ORF10 had a total of 92, 8, 54, 33 and 17 nucleotide-level variations, respectively whereas 105, 158 and 6-nt mutations were sequentially detected in 5´-UTR, 3´-UTR, and spacer regions (Supplementary Table 1b).

Moreover, in the ORFs regions total 1,247 nt mutations were observed and among them 503 were missense mutations. In the ORF1ab polyprotein, 120, 33, 57, 44 and 11 aa substitutions have been identified in the NSP3, NSP4, NSP2, NSP12 and NSP5, respectively (Supplementary Table 1b). In case of spike protein, 11 aa substitutions (V341I, A348T, N354D, D364Y, V367F, K378R, Q409E, A435S, G476S, V483A, and Y508H) have been found in the receptor-binding domain (RBD) at 331 to 524 residues of S1 subunit in Wales, USA, Shenzhen, Shenzhen, Hong Kong/France, Shanghai, Guangdong, Finland, USA, USA, and France, respectively, among which three substitutions occurred in the positions between 424 to 494 comprising the receptor-binding motif (RBM). Sarkar et al. (2020) identified a unique mutation in the S protein (A930V)^8^ in the Indian SARS-CoV-2, which was absent in other related SARS-CoV-2 strains of different geographical regions. Additionally, we identified twelve and five aa replacements in the Heptad Repeat-1 and 2 (HR1, HR2) comprising 892–1,013 and 1,145–1,195 positions in the S2 subunit, respectively. We observed 5 aa replacements in the M protein in the topological domains (1–18 and 71–78), and the E and N proteins had 10 and 75 aa replacements, respectively.

In a recent study, Yuan et al. demonstrated the molecular insights of a cross-reacting neutralizing antibody CR3022 to the highly conserved cryptic epitope^23^ (86% conserved between SARS-CoV and SARS-CoV-2) on the receptor-binding domain (RBD) of the SARS-CoV-2 spike protein, but distal to the receptor-binding site linked to the angiotensin-converting enzyme 2 (ACE2) of host cell surface during infecting the cells. Significantly, our results identified one aa substitution (K378R) among the 28 residues of that epitope in hCoV-19/Shanghai/SH0007/2020 (EPI_ISL_416320) strain, which was isolated on 28 January, 2020 from China. The aa residues (D54 and E56) on the paratope of antibody interact with the lysine situated on the epitope, however the replaced arginine found in the strain hCoV-19/Shanghai/SH0007/2020 might have a superior binding to the neutralizing antibody because of stronger electrostatic interactions, such as salt-bridges and hydrogen bonds compared to lysine^24^. At this point, we must stress that the changing of this aa is not directly linked to immunological pressure after applying the convalescent plasma therapy on the patients of China started from 9 February 2020, rather can be an evolutionary purifying selection possibly increasing stronger interaction with antibody^25^. Noteworthy, this significant mutation at position 378 was not found in other virus strains analyzed in this study. Thus, it might be of evolutionary selection or due to sequence error in this particular strain. However, further investigation into the newly produced sequences needs to be confirmed about this mutation. In addition, it should be mentioned that the residues associated with ACE2 binding to the spike protein (439N, 449Y, 453Y, 455L, 456F, 475A, 486F, 487N, 489Y, 493Q, 495Y, 498Q, 499T, 500N, 501G, 504Y) have not shown any variation in the mutational analyses^23^.

### Deletion mutations in both coding and non-coding sequences

In addition to site-specific mutations, we found 12 deletion-sites of ranged nucleotides in polyprotein (n = 9; NSP1:6, NSP2:1, NSP8:1, NSP15:1), ORF10 (n = 1) and 3´-UTR (n = 2) in 120 strains reported from Japan, USA, England, Canada, Netherlands, China, Australia, Spain, Portugal, Slovakia, Iceland, Wales, France and New Zealand (Fig. 1, Supplementary Table 1c). We screened out the established deletions (ORF8∆381, ORF7a∆81 and spike∆15) within the SARS-CoV-2 genomes since they were already reported^17,26,27^, and notably, in vivo experiment using spike protein deletion variant (∆15 at S1-S2 junction) was also performed^27^. The 382, 81 and 15-nt deletion of ORF8, ORF7a and spike protein, respectively, have been predicted to affect the viral adaptation to human, virus-host interactions for infections, attenuation, pathogenicity, and immune-modulations by potentially influencing the tertiary structures and functions of the associated proteins^5,28–30^. On this note, Liu et al. (2020) identified two previously unreported common deletions with a very low frequency at 23,585–23,599 (aa-QTQTN)^26^ positioned at the upstream of the polybasic cleavage site of S1–S2, and 23,596–23,617 (aa-NSPRRAR) including the polybasic cleavage site in the clinical samples and cell-isolated virus strain^31^. In addition, three SARS-CoV-2 strains (Singapore/12-14/2020|EPI_ISL_414378-80) reported earlier from Singapore with an impact of enhanced transcription of the subsequent N protein^32^ indicating ongoing evolution of the virus. Therefore, we acknowledged these established works and further checked the other deletions alongside their possible roles.

Here, we spotted six deletions (∆24:94–117, ∆15:243–257, ∆9:421–429, ∆3:253–255, ∆3: 404–406 and ∆3:251–253) in the NSP1 (host translation inhibitor) (Fig. 1a–c, Supplementary Table 1c). To our knowledge and based on this dataset, the 24-nt deletion (G32:L39) is only found in Japanese strain^5,33^. Remarkably, other deletions within NSP1 have not been reported elsewhere, and are found in 16 virus strains of USA, Canada, Japan, England, Iceland and Netherlands collected until 14th March, 2020. Further *in-silico* and wet lab experiments are necessary to investigate the appropriate role of the NSP1 deletion variants in the replication and pathogenesis.

Since the deletion of NSP2 (1,340–1,342: D268) corroborated with the previous studies^17,26,27^, we modelled (data not shown) and found that the aspartic acid situated on the protein surface signifying its probable role in the viral pathogenesis^19^. Furthermore, Bal et al. (2020) identified 37 deleted virus strains collected in England (February) and in the Netherlands (March)^17^ whereas we here report 99 such strains from the Netherlands (59), England (21), Portugal (2), Slovakia (1), Iceland (5), Wales (8), France (2), and New Zealand (1) suggesting the spread of this deletion mainly in Europe, especially in the Netherlands (Fig. 1d, Supplementary Table 1c). Another co-linked three nt deletion at 12,365-67 (∆Ser177) of NSP8 (primase activity) was found in only two strains of Netherlands (Netherlands/NoordBrabant_49 and 52/2020) (Fig. 1e, Supplementary Table 1c). It is important to mention here that NSP1 deletion variants ∆15:243–257 and ∆3:253–255 are coexisting with two NSP2 deletion variant strains, collected in the Netherland (Netherlands/NoordBrabant_31 and 54/2020) as well. Therefore, these deletions might be co-evolving and may have specific roles in viral replication.

Strikingly, the deletion of the initial protein coding portions, including the initial first 21-nt from the ORF10, and probable usage of a different start codon is of particular interest. Only two strains from Madrid, Spain (EPI_ISL_417956 and EPI_ISL_417957 have such frameshifted deletion and alternative codon usage capacity. Remarkably, ORF10 undergoes a deletion (35 nt) including its start codon, and instead, the start codon of adjacent spacer region can probably be used for protein coding (Fig. 1g). Notably, the specific functions of the accessory proteins ORF10 remained mysterious. Nevertheless, recent *in-silico* analyses predicted the antagonistic attacking of ORF10 protein to the heme that dissociates the iron from porphyrin. These effects are linked to the disease manifestations and clinical outcomes of the patients; hence the deletions may have possible roles in virulence and pathogenicity^31^.

Less significantly, 3 nt deletion (20,031–20,033: ∆Leu119 within the middle domain) in the uridylate-specific endoribonuclease or NendoU, NSP15, as reported earlier^5,18^, is also found in the same US strain (USA/WI1/2020) (Fig. 1f, Supplementary Table 1c). Interestingly, we report a novel 29-nt deletion in a strain from Wuhan (Fig. 1h) and another established 10-nt deletion in the Australian strain (Fig. 1i) within the 3′-UTR. The deletions at the 3′-UTR may cause forming of different pseudoknots, hence have implications in viral replication and recruiting host translational machinery by the stem-loop II-like motif (s2m), and in parallel, previous report^34^ also suggested in vivo functional roles of 3′-UTR of SARS-CoV-2. The associations between/among the deletions and other factors like climate, mortality and geography cannot be explained because of very less members of randomized deletions observed in genome sequences of each country. However, one interesting fact was observed in case of Netherlands, finding 62 deletions out of total 123 reported in this study. Nevertheless, any predicted attenuation roles of those Dutch deletions will be misleading because of relative population size of the European countries where more than 10,000 infected cases were reported, such as England, Italy, Portugal, Belgium and France.

### Point-specific amino-acid replacements: significant heterogeneity observed in S, E, and M proteins

**Figure 2:**
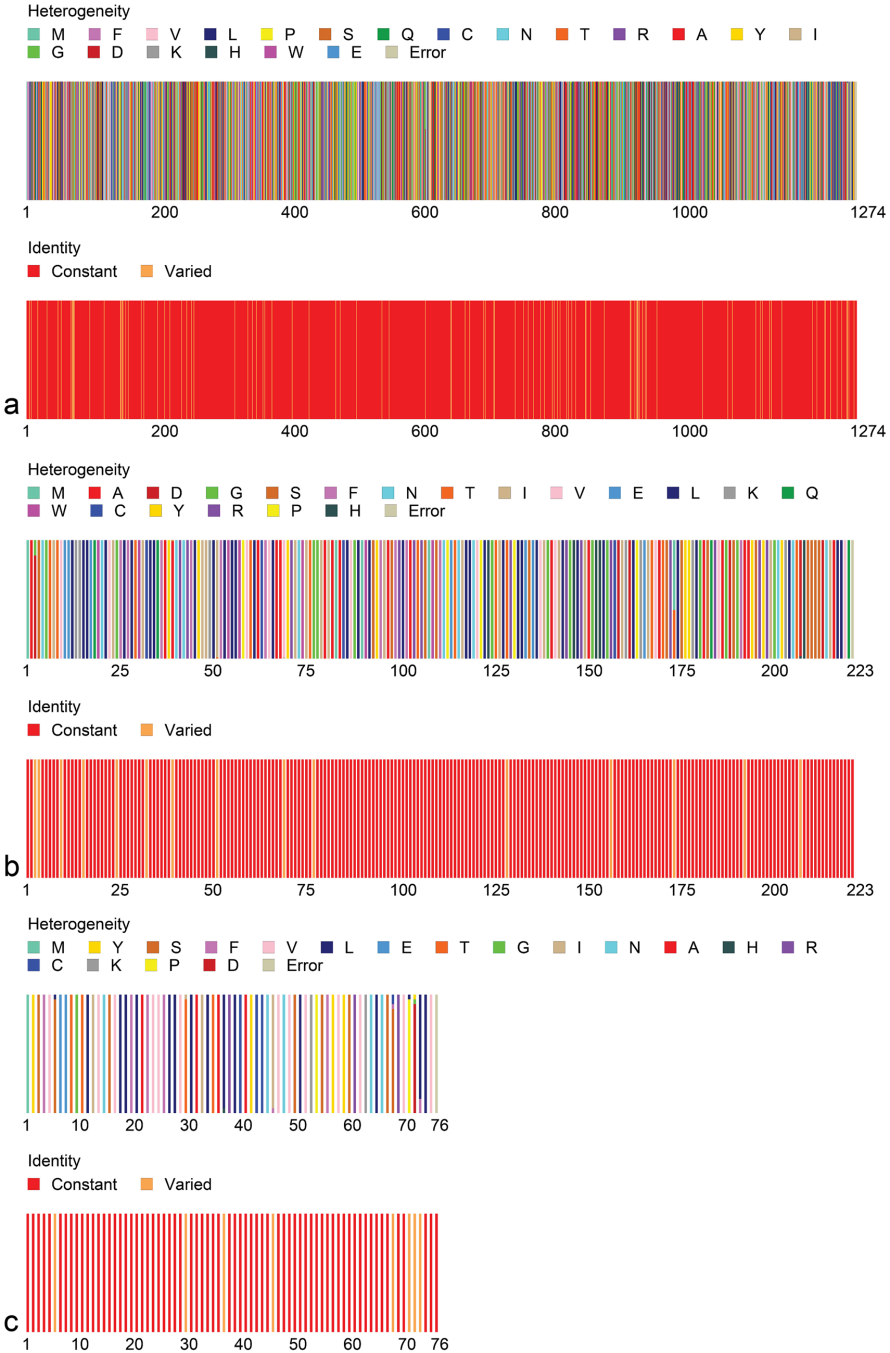
Amino-acid (aa) residues heterogeneity in (**a**) S, (**b**) E and (**c**) M proteins of SARS-CoV-2. The Fingerprint^39^ protein analysis showed that aa residues in S, M and E proteins varied due to change and/or substitutions in their positions.

Another noteworthy finding of our present systematic analysis is the identification of 744 aa replacements at different positions of the entire SARS-CoV-2 proteomes due to nonsynonymous mutations in SAR-CoV-2 genomes. We found 412 aa replacements in polyproteins ORF1ab of SARS-CoV-2 and 228 in the structural proteins comprising 120, 15, 11 and 82 positions in S, M, E, and N proteins, respectively. Besides structural proteins, 48, 5, 25, 16 and 10 aa replacements were identified in the accessory proteins, ORF3a, ORF6, ORF7ab, ORF8, and ORF10, respectively (Supplementary Table 1b). The protein sequence heterogeneity profile obtained from Fingerprint analysis revealed significant heterogeneity in the aa residues within immunologically important structural proteins, S, E and M of different strains of SARS-CoV-2 (Fig. 2). Also, the high morbidity and mortality of immune-compromised patients infected by COVID-19 might be correlated with the disastrous consequences of these mutations^5,11^.

### Geo-climate patterns and severity of COVID-19: a possible link with viral evolution

**Figure 3:**
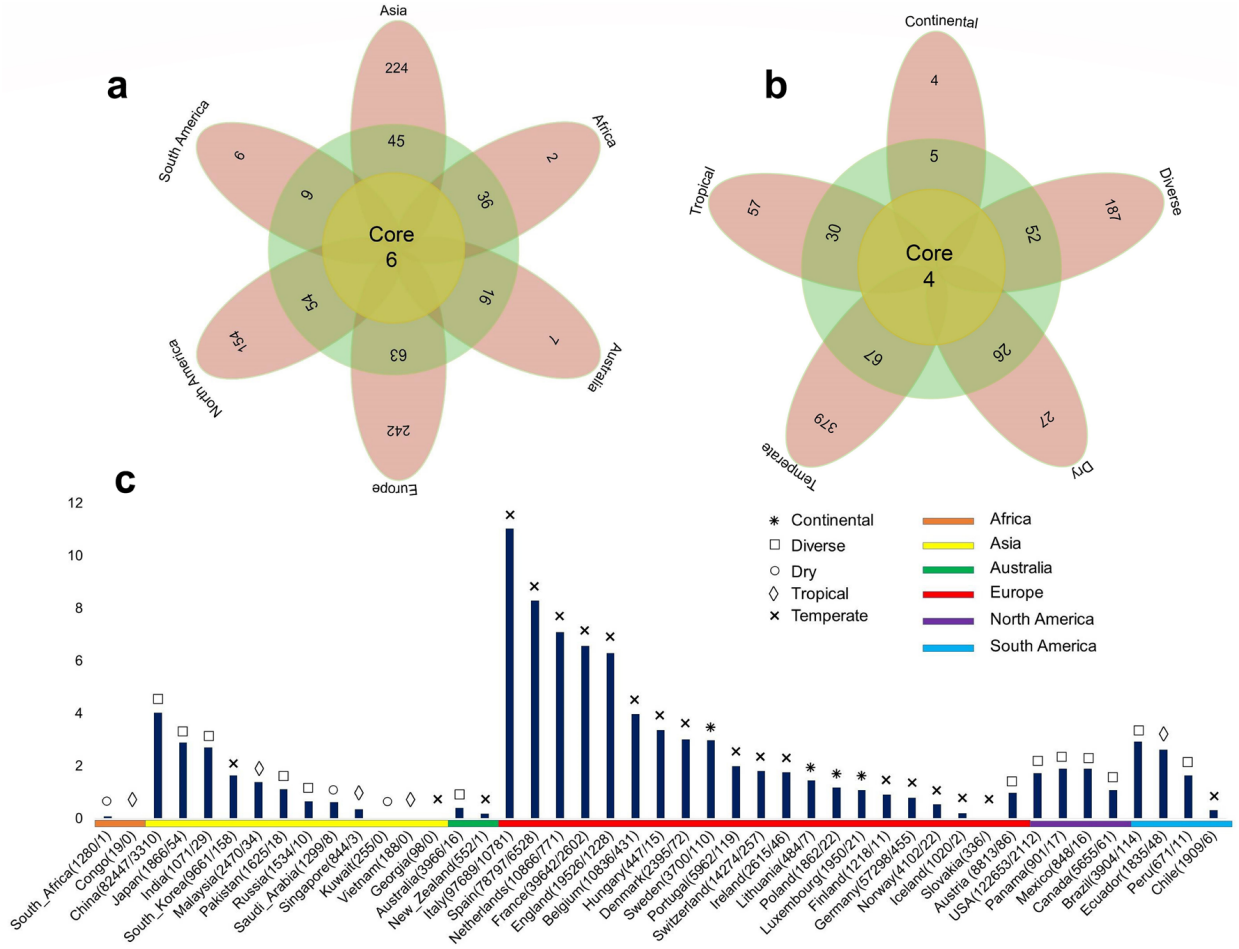
The frequency spectra of amino-acid mutations in 2,492 SARS-CoV-2 complete genome sequences, and its impact on mortality rates. Amino-acid (aa) mutations found in the open reading frames (ORFs) of the SARS-CoV-2 genomes according to (**a**) geographic areas and (**b**) different climate zones. We found six core shared mutations (at residue position of R203K, G204R, G251V, L3606F, P4714L, D614G) in Asia, Europe, Africa, Australia, North America, and South America, and four core shared mutations (at residue position of Q57H, D614G, L3606F, and P4714L) in continental, diverse, dry, tropical and temperate conditions. In both cases (**a** and **b**), yellow circles represent frequency of aa substitutions shared by all categories, and the frequency of aa substitutions shared by at least two continents/climate zones are shown in green circle, while the pink colored ribbons indicate unique aa mutations in each individual regional and climate zone. (**c**) Estimated case fatality rates of SARS-CoV infections in 45 countries of the globe according to climatic variations. Numbers in parentheses indicate total confirmed cases/total deaths according to the World Health Organization (WHO) as of 30th March, 2020. Organization (WHO) as of 30th March, 2020. Data were finally visualized using custom Venn diagrams online tool (https://bioinformatics.psb.ugent.be/webtools/Venn/) along with illustrator CC 2019 trial version.

With the march of time, more and more missense mutations are being detected among the genome sequences of SARS-CoV-2. In addition to global nt mutations in genomes and 744 aa changes in proteomes were detected having mutations from 2,492 complete (and/or near complete) genome sequences of SARS-CoV-2. We also found that aa changes patterns are different among the genomes of SARS-CoV-2 in six continents. It seems that aa changes were relatively higher in the SARS-CoV-2 sequences of Europe (43.07%) followed by Asia (38.09%), North America (29.64%), and rest of the continents (Africa, Australia and South America) had a relatively lowered frequency (< 1.0%) (Fig. 3a, Supplementary Data 1). Of the detected mutations, only six (< 1.0%) core aa substitutions (R203K, G204R, G251V, L3606F, P4714L, D614G) across open reading frames (ORFs) the SARS-CoV-2 genomes found in all the six continents (Fig. 3a). However, 242, 224, 154, 7, 6, and 2 unique aa replacements, and 63, 54, 45, 36, 16, and 6 accessory replacements (shared with at least two continents) were found in the SARS-CoV-2 genomes sequenced from Europe, Asia, North America, Australia, South America, and Africa, respectively (Fig. 3a, Supplementary Data 1). Higher unique mutations in European, Asian and North American sequences indicate the geographically clustering tendency of the virus. Further phylogenic study targeting those unique mutations may guide to proper global phylodynamics of the virus and modulate the regional control strategy against the COVID-19 pandemic.

Simultaneously, we found that nonsynonymous mutations also varied according to different climatic conditions. In this study, only four core aa substitutions (Q57H, D614G, L3606F, and P4714L) were found across all the climatic zones (Fig. 3b). Notably, three replacements L3606F, P4714L, D614G found in all geographic and climatic zones indicating their special importance in universal infectivity and whole virus vaccine development for world population. Conversely, 379,187, 57, 27, and 4 unique nonsynonymous mutations were found in the SARS-CoV-2 genomes belonged to temperate, diverse, tropical, dry and continental climatic conditions, respectively. Moreover, 66, 52, 30, 26, and 5 residues positioned where mutations occurred at least in two climatic zones (Fig. 3b, Supplementary Data 1). Temperate European countries such as Italy, Spain, France, Netherlands and England are major COVID-19 infected countries with higher mortality rates at this initial stage. These finding are in line with Deshwal^30^ who also reported highest SARS-CoV-2 infections and case fatality rates in European countries. Besides, two previously reported nonsynonymous mutations (R203K and L3606F)^11^ were shared across ORFs of the SARS-CoV-2 genomes of six continents, and co-occurrence of those mutations were also common in different countries along with unique mutations. However, these unique or accessory mutations are driven by the geographic locations that may have impact on differntial divergence of the virus strains, possibly due to the changed environment, varied demography, the lower fidelity of the RNA dependent RNA polymerase (RdRp), and/or relatively less efficient proof-reading activity of the NSP14^11,21,22^.

By comparing the death severity rates of SARS-CoV-2 infections in 45 countries across the globe according to different climatic conditions, we found that most of the temperate countries had higher mortality rates than other climatic zones (Fig. 3c). Among the temperate countries, highest mortality rate was found in Italy (11.04%) followed by Spain (8.29%), Netherlands (7.08%), France (6.56%), England (6.29%), China (4.02%), Belgium (3.98%), Hungary (3.36%), Denmark (3.01%), and rest of the countries had less than 3.0% SARS-CoV-2 case fatality rates (Fig. 3c, Supplementary Data 1). The death rates from SARS-CoV-2 infections greatly varied in diverse climatic conditions of Brazil, Pakistan, and Australia, where we found 2.92%, 1.11%, and 0.4% death rates, respectively. Similarly, in tropical climate conditions of India, Ecuador, Panama, Mexico, Peru, and Malaysia, we found 2.71%, 2.62%, 1.89%, 1.89%, 1.64%, and 0.38% mortality rates, respectively. The continental countries, USA and Lithuania had observed mortality rates of 1.72%, and 1.45%, respectively from SARS-CoV-2 infections. However, mortality rates in dry countries like Saudi Arabia were much lower (< 1.0%) compared to other climatic conditions (Fig. 3c, Supplementary Data 1). Comparatively higher mortality rates in European temperate countries might be correlated with higher unique mutations found in the viruses reported from this geo-climate region. Despite higher unique mutations in Asian and North American strains, these regions showed less case fatality rates compared to the European countries. These findings predicted the European unique mutations to be associated with higher pathogenicity of the virus. However, it is worth noting that reported disease severity (may not represent the actual severity) might be affected by several other factors, for example, health care facilities, average age group, genetic context of the population and control strategies adopted by the countries. Regardless of the importance of geography on the COVID-19 epidemiology, the effects of global mobility upon the genetic diversity and molecular evolution of SARS-CoV-2 are underappreciated and only beginning to be comprehended. Moreover, the recent monograph on the spatial epidemiology of COVID-19 makes no reference to the genetic variation of SARS-CoV-2^20–22^.

The limitations we faced in this study are due to the nature of the SARS-CoV-2 genomic data where the sample collection dates might not reveal the actual infection dates, and not all countries faced SARS-CoV-2 infections have timely uploaded the sequences to the GISAID database. Thus, the mutation patterns might be an approximate finding. Moreover, many countries have not sequenced enough virus samples (such as African and Sub-Saharan countries), and some countries uploaded sequences collected from samples of single-source or zone of infection (Japan), hence the mutation pattern may be biased in specific country or continent. Nevertheless, our study had included the most complete available SARS-CoV-2 sequences up to March 30, 2020. This study, therefore, opens up new perspectives to determine whether one of these frequent mutations will lead to biological differences, and their correlation with different case fatality rates.

## Conclusions

This study reveals a number of unreported mutations, which cover both mismatches and deletions in translated and untranslated regions of the SARS-CoV-2 genomes. Moreover, the geo-climate distribution of the mutations deciphered higher unique mutations as well as disease severity in the European temperate countries. Further investigations should focus on structural validations and subsequent phenotypic consequences of the deletions and/or mismatches in transmission dynamics of the current epidemics and the immediate implications of these genomic markers to develop potential prophylaxis and mitigation for tackling the crisis of pandemic COVID-19. Moreover, the identification of the conformational changes in mutated protein structures and untranslated cis-acting elements is of significance for studying the virulence, pathogenicity and transmissibility of SARS-CoV-2. This mutational diversity should be investigated by further studies, including their metabolic functional pathway, intra-viral and virus-host interactions analyses.

## Methods

### Sequence retrieval and alignment

To decipher the genetic variations, we retrieved two thousand four hundred and ninety-two (n = 2,492) complete or near-complete genomes of SARS-CoV-2 available at the global initiative on sharing all influenza data (GISAID) (https://www.gisaid.org/) up to 30 March 2020. We divided the SARS-CoV-2 genome sequence data according to their geographic origins from six continents such as Europe, Asia, North America, South America, Africa, and Australia, and five related climatic zones including temperate, tropical, diverse, dry and continental. To estimate the case fatality (mortality) rates of SARS-CoV-2 infections, we collected information on total infected cases, and total reported deaths in these countries from the World Health Organization (WHO) COVID-19 Reports^35^ up to March 30, 2020. These SARS-CoV-2 sequences belonged to the infected patients from 58 countries of six continents (Supplementary Fig. 1, Supplementary Table 1a). We aligned the SARS-CoV-2 genome sequences using MAFFT online server^36^ using default parameters, and the complete genome sequence SARS-CoV-2 Wuhan-Hu-1 strain (Accession NC_045512, Version NC_045512.2) was used as a reference genome.

### Mutation analysis

We used MEGA 7^37^ for multiple sequence alignments to differentiate the SARS-CoV-2 genomes according to their open reading frames (ORF). Sequence cleaner (https://github.com/metageni/Sequence-Cleaner) was used to remove all ambiguous and low-quality sequences. SeqKit^38^ toolkit was used to intercept gap containing strains for deletion analysis. Internal stop codon containing sequences were removed by using SEquence DAtaset builder (SEDA; https://www.sing-group.org/seda/). Amino-acid heterogeneity analysis was performed with Fingerprint, a web-based protein profile analysis tool^39^. Amino-acid mutation analysis was done by simple bio-python program with pairwise alignment (https://github.com/SShaminur/Mutation-Analysis). We used custom Venn diagrams (https://bioinformatics.psb.ugent.be/webtools/Venn/) server to create the Venn diagrams. Finally, the aligned sequences were visualized using Unipro-UGENE 1.26.1 to visualize the deletions with respect to the reference genome^40^.

## Supplementary information

Supplementary Data 1.

Supplementary Figures.

Supplementary Tables.

Acknowledgements file.

## Data Availability

We used the complete genome sequence SARS-CoV-2 Wuhan-Hu-1 strain (Accession NC_045512, Version NC_045512.2) as a reference genome. The annotated genomes (n = 2,492) have been retrieved from publicly open database, the global initiative on sharing all influenza data (GISAID) (https://www.gisaid.org/) up to March 30, 2020. Further information regarding the accession numbers of the studied genomes could be found in the Supplementary Data 1.
