# Author Correction: Genome-wide analysis of SARS-CoV-2 virus strains circulating worldwide implicates heterogeneity

**DOI:** 10.1038/s41598-021-00133-9

**Published:** 2021-10-12

**Authors:** M. Rafiul Islam, M. Nazmul Hoque, M. Shaminur Rahman, A. S. M. Rubayet Ul Alam, Masuda Akther, J. Akter Puspo, Salma Akter, Munawar Sultana, Keith A. Crandall, M. Anwar Hossain

**Affiliations:** 1 Department of Microbiology, University of Dhaka, Dhaka, 1000 Bangladesh; 2 Department of Gynecology, Obstetrics and Reproductive Health, Bangabandhu Sheikh Mujibur Rahman Agricultural University, Gazipur, 1706 Bangladesh; 3 Department of Microbiology, Jashore University of Science and Technology, Jashore, 7408 Bangladesh; 4 Department of Microbiology, Jahangirnagar University, Savar, Dhaka, 1342 Bangladesh; 5 Computational Biology Institute, Milken Institute School of Public Health, The George Washington University, Washington, USA; 6 Present Address: Jashore University of Science and Technology, Jashore, 7408 Bangladesh

Correction to: *Scientific Reports* 10.1038/s41598-020-70812-6, published online 19 August 2020

The Acknowledgments section in the original version of this Article was incomplete.

“The authors of this manuscript would like to extend their thank to all who have contributed sequences to the GISAID database (https://www.gisaid.org/).”

now reads:

“The authors of this manuscript would like to extend their thanks to all who have contributed sequences (Acknowledgements File) to the GISAID database (https://www.gisaid.org/).”

The original Article and accompanying Supplementary Information files have been corrected.

