# Supplementary material for: Genome-wide analysis of SARS-CoV-2 virus strains circulating worldwide implicates heterogeneity
Source: Sci Rep. 2020 Aug 19;10:14004. doi: 10.1038/s41598-020-70812-6 (PMC7438523; doi:10.1038/s41598-020-70812-6)
Supplement: Supplementary file 2 — Supplementary Figures. [file 41598_2020_70812_MOESM2_ESM.docx]

**Supplementary Figure**

| 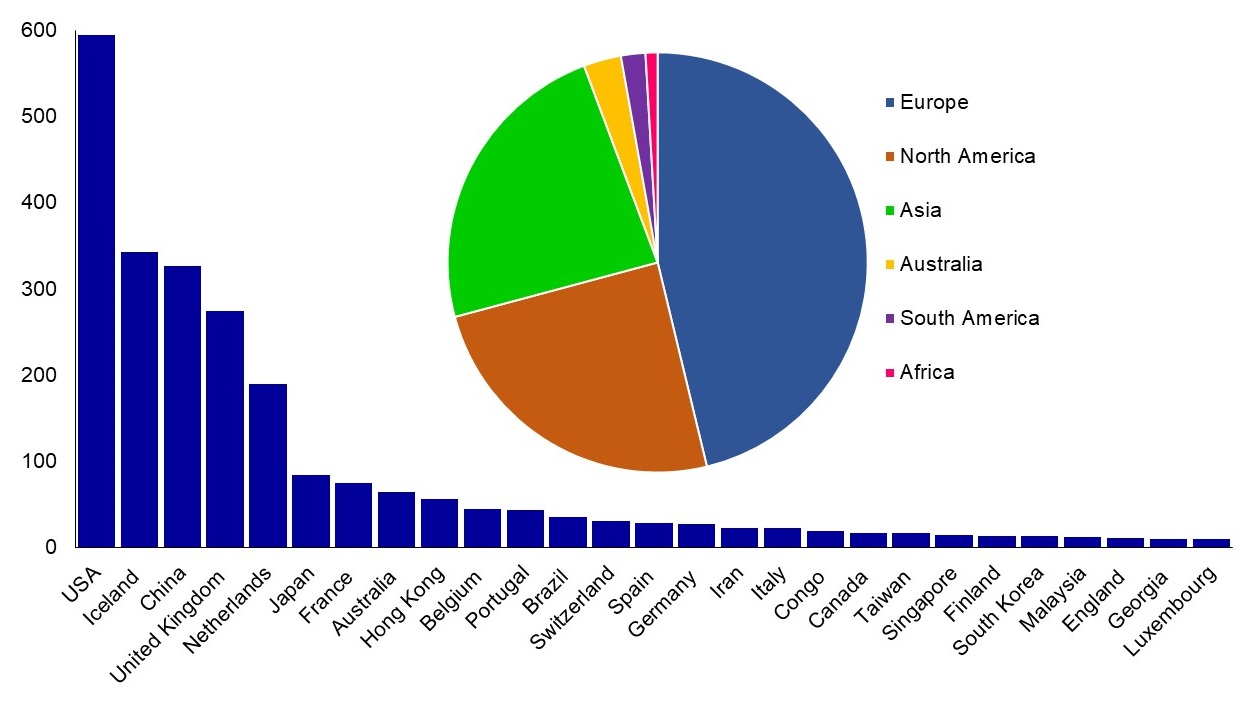 |
| --- |

**Supplementary Fig. 1** **Geographic distribution of SARS-CoV-2 strains**. We retrieve 2,492 SARS-CoV-2 sequences from the GISAID belonged to the infected patients from 58 countries across six continents. Countries having > 20 sequences are depicted in bar plot, and rest are available in Supplementary Table 1a.
