# Supplementary material for: Genome-wide analysis of SARS-CoV-2 virus strains circulating worldwide implicates heterogeneity
Source: Sci Rep. 2020 Aug 19;10:14004. doi: 10.1038/s41598-020-70812-6 (PMC7438523; doi:10.1038/s41598-020-70812-6)
Supplement: Supplementary file 3 — Supplementary Tables. [file 41598_2020_70812_MOESM3_ESM.docx]

**Genome wide analysis of Severe Acute Respiratory Syndrome Coronavirus-2 implicates world-wide circulatory virus strains heterogeneity**

M. Rafiul Islam, M. Nazmul Hoque, M. Shaminur Rahman, A. S. M. Rubayet Ul Alam, Masuda Akther, J. Akter Puspo, Salma Akter, Munawar Sultana, Keith A. Crandall, M. Anwar Hossain

**Supplementary Table 1a: Geographical distribution of the sequences**

| Country | Number of sequences |
| --- | --- |
| USA | 595 |
| Iceland | 343 |
| China | 327 |
| United Kingdom | 275 |
| Netherlands | 190 |
| Japan | 84 |
| France | 75 |
| Australia | 64 |
| Hong Kong | 56 |
| Belgium | 45 |
| Portugal | 44 |
| Brazil | 35 |
| Switzerland | 31 |
| Spain | 28 |
| Germany | 27 |
| Iran | 23 |
| Italy | 22 |
| Congo | 19 |
| Canada | 17 |
| Taiwan | 17 |
| Singapore | 14 |
| Finland | 13 |
| South Korea | 13 |
| Malaysia | 12 |
| England | 11 |
| Georgia | 10 |
| Luxembourg | 10 |
| Denmark | 9 |
| New Zealand | 8 |
| Norway | 8 |
| Chile | 7 |
| Ireland | 6 |
| Vietnam | 6 |
| Indonesia | 4 |
| Kuwait | 4 |
| Philippines | 4 |
| Slovakia | 4 |
| Czech Republic | 3 |
| Hungary | 3 |
| India | 3 |
| Saudi Arabia | 3 |
| Peru | 2 |
| Senegal | 2 |
| Thailand | 2 |
| Cambodia | 1 |
| Colombia | 1 |
| Ecuador | 1 |
| Lithuania | 1 |
| Mexico | 1 |
| Nepal | 1 |
| Nigeria | 1 |
| Pakistan | 1 |
| Panama | 1 |
| Poland | 1 |
| Russia | 1 |
| South Africa | 1 |
| Sweden | 1 |
| Turkey | 1 |
| Continent-wise |  |
| Continent | Number |
| Europe | 1152 |
| North America | 613 |
| Asia | 585 |
| Oceania | 72 |
| South America | 46 |
| Africa | 23 |
| Central America | 1 |
| Total | 2492 |

**Supplementary Table 1b: Summary of genomic analysis**

| **Saummary of genomic analysis** | |
| --- | --- |
|  |  |
| Total nucleotide change (mutations) | 1516 |
| Coding region | 1247 |
| Non-coding region | 269 |
| Total synonymus mutations | 503 |
| Total amino acid change or substitution | 744 |
|  |  |
| **Region** | **Amino Acid Mutation Number** |
| Polyprotein | 412 |
| S | 120 |
| M | 15 |
| E | 11 |
| N | 82 |
| ORF3a | 48 |
| ORF6 | 5 |
| ORF7a | 22 |
| ORF7b | 3 |
| ORF8 | 16 |
| ORF10 | 10 |
| **Polyprotein** | **Amino Acid Mutation Number** |
| nsp1 | 11 |
| nsp2 | 57 |
| nsp3 | 120 |
| nsp4 | 33 |
| nsp5 | 11 |
| nsp6 | 16 |
| nsp7 | 2 |
| nsp8 | 6 |
| nsp9 | 5 |
| nsp10 | 6 |
| nsp12 | 44 |
| nsp13 | 31 |
| nsp14 | 27 |
| nsp15 | 30 |
| nsp16 | 13 |
|  |  |
| **Region** | **Nucleotide Mutation Number** |
| Polyprotein | 661 |
| S | 183 |
| M | 34 |
| E | 17 |
| N | 148 |
| ORF3a | 92 |
| ORF6 | 8 |
| ORF7a | 46 |
| ORF7b | 8 |
| ORF8 | 33 |
| ORF10 | 17 |

|  | **Polyprotein** | | |
| --- | --- | --- | --- |
|  |  |  |  |
|  |  |  |  |
| **Non-strictural protein** | **Country** | **Amino acid change (ref:position:strain)** | **Total Nucleotide mutations** |
| nsp1 | Shanghai | G7V | 661 |
|  | USA | V23F |  |
|  | Japan | R24C |  |
|  | Portugal | R24C |  |
|  | USA | G30D |  |
|  | Malaysia | H45Y |  |
|  | England | V56I |  |
|  | Wales | V56I |  |
|  | New_Zealand | D75E |  |
|  | USA | D75E |  |
|  | Iceland | E87D |  |
|  | Belgium | L104F |  |
|  | USA | A117T |  |
|  | France | Y118C |  |
|  | Luxembourg | Y118C |  |
| nsp2 | USA | F190L |  |
|  | Iceland | P193S |  |
|  | Australia | R207C |  |
|  | Australia | R207C |  |
|  | Canada | R207C |  |
|  | Kuwait | R207C |  |
|  | Pakistan | R207C |  |
|  | Slovakia | R207C |  |
|  | Georgia | E217G |  |
|  | USA | T224I |  |
|  | Belgium | T265I |  |
|  | Denmark | T265I |  |
|  | Finland | T265I |  |
|  | France | T265I |  |
|  | Georgia | T265I |  |
|  | Germany | T265I |  |
|  | Iceland | T265I |  |
|  | Luxembourg | T265I |  |
|  | NanChang | T265I |  |
|  | Netherlands | T265I |  |
|  | Portugal | T265I |  |
|  | Taiwan | T265I |  |
|  | USA | T265I |  |
|  | Australia | N272H |  |
|  | France | P309S |  |
|  | USA | P309L |  |
|  | Iceland | S318L |  |
|  | USA | H325Y |  |
|  | Denmark | T346I |  |
|  | Iceland | T346I |  |
|  | Iceland | E352D |  |
|  | USA | A354V |  |
|  | Iceland | T356A |  |
|  | Georgia | H374Y |  |
|  | USA | H374Y |  |
|  | Australia | V378I |  |
|  | Canada | V378I |  |
|  | England | V378I |  |
|  | Germany | V378I |  |
|  | Kuwait | V378I |  |
|  | Netherlands | V378I |  |
|  | Pakistan | V378I |  |
|  | Shandong | V378I |  |
|  | Shanghai | V378I |  |
|  | Taiwan | V378I |  |
|  | USA | V378I |  |
|  | USA | A385V |  |
|  | Guangdong | N389D |  |
|  | Guangzhou | N389D |  |
|  | Australia | G392D |  |
|  | Brazil | G392D |  |
|  | Germany | G392D |  |
|  | Iceland | G392D |  |
|  | Netherlands | G392D |  |
|  | Poland | G392D |  |
|  | USA | G392D |  |
|  | Wales | G392D |  |
|  | Iceland | R398C |  |
|  | Netherlands | R398C |  |
|  | USA | R402C |  |
|  | Belgium | G408S |  |
|  | Australia | H417R |  |
|  | Belgium | H417R |  |
|  | England | H417R |  |
|  | England | H417Y |  |
|  | Iceland | H417R |  |
|  | Scotland | H417R |  |
|  | USA | H417R |  |
|  | USA | A427V |  |
|  | France | S428G |  |
|  | USA | S428N |  |
|  | Hong_Kong | E444K |  |
|  | India | I476V |  |
|  | South_Korea | K492T |  |
|  | South_Korea | S505F |  |
|  | Shanghai | G514R |  |
|  | Australia | G519S |  |
|  | Iceland | G519S |  |
|  | Iceland | A537T |  |
|  | USA | A555V |  |
|  | USA | R560H |  |
|  | France | L580F |  |
|  | Iceland | M585V |  |
|  | England | T609I |  |
|  | USA | T609I |  |
|  | USA | W630R |  |
|  | Kuwait | E658D |  |
|  | India | I671T |  |
|  | Shanghai | Q676H |  |
|  | France | L681F |  |
|  | China | A690V |  |
|  | Brazil | T708I |  |
|  | Australia | T727I |  |
|  | Belgium | G728S |  |
|  | USA | A735V |  |
|  | Australia | I739V |  |
|  | Congo | I739V |  |
|  | England | I739V |  |
|  | Iceland | I739V |  |
|  | Netherlands | I739V |  |
|  | Portugal | I739V |  |
|  | Scotland | I739V |  |
|  | Netherlands | P748S |  |
|  | Netherlands | E754D |  |
|  | Australia | P765S |  |
|  | Congo | P765S |  |
|  | England | P765S |  |
|  | Iceland | P765S |  |
|  | Netherlands | P765S |  |
|  | Portugal | P765S |  |
|  | Scotland | P765S |  |
|  | Shanghai | P769S |  |
|  | USA | M789I |  |
|  | USA | P804L |  |
|  | Sweden | G818S |  |
| nsp3 | Iceland | V839L |  |
|  | Iceland | I841V |  |
|  | Australia | A876T |  |
|  | Brazil | A876T |  |
|  | Germany | A876T |  |
|  | Iceland | A876T |  |
|  | Netherlands | A876T |  |
|  | Poland | A876T |  |
|  | USA | A876T |  |
|  | Wales | A876T |  |
|  | Scotland | P885S |  |
|  | Iceland | P892S |  |
|  | South_Korea | M902I |  |
|  | Iceland | A903V |  |
|  | South_Korea | F941L |  |
|  | USA | T945I |  |
|  | Japan | Q956R |  |
|  | Netherlands | L960F |  |
|  | Shandong | L969P |  |
|  | France | P971S |  |
|  | New_Zealand | P971L |  |
|  | Shanghai | P971S |  |
|  | USA | P971L |  |
|  | Tianmen | E972K |  |
|  | USA | G993S |  |
|  | USA | Q998H |  |
|  | England | D1036E |  |
|  | Portugal | D1036E |  |
|  | Chile | A1043V |  |
|  | USA | E1047D |  |
|  | USA | A1049V |  |
|  | USA | L1110F |  |
|  | USA | K1124E |  |
|  | France | G1155C |  |
|  | Australia | A1156V |  |
|  | USA | P1158L |  |
|  | Jiangsu | T1168I |  |
|  | Japan | A1176V |  |
|  | Iceland | S1189G |  |
|  | Japan | K1197E |  |
|  | Jiangsu | V1199I |  |
|  | USA | K1202N |  |
|  | Hangzhou | P1207H |  |
|  | Shanghai | P1207H |  |
|  | USA | K1230N |  |
|  | France | T1241I |  |
|  | Denmark | T1246I |  |
|  | England | T1246I |  |
|  | Iceland | T1246I |  |
|  | Portugal | G1261D |  |
|  | Iceland | I1286M |  |
|  | Japan | G1288S |  |
|  | USA | V1295F |  |
|  | Japan | A1298V |  |
|  | Japan | M1312I |  |
|  | USA | N1324S |  |
|  | Luxembourg | A1352V |  |
|  | Shanghai | S1400P |  |
|  | USA | K1410R |  |
|  | USA | A1420T |  |
|  | Shanghai | T1429A |  |
|  | Shanghai | T1444I |  |
|  | Germany | R1464W |  |
|  | Shanghai | R1464W |  |
|  | USA | A1473V |  |
|  | USA | P1497S |  |
|  | USA | P1497L |  |
|  | USA | I1505T |  |
|  | Russia | N1576T |  |
|  | USA | P1596L |  |
|  | Beijing | L1599F |  |
|  | South_Korea | L1599F |  |
|  | Canada | I1607V |  |
|  | Shanghai | I1607V |  |
|  | USA | I1607V |  |
|  | Iceland | T1626I |  |
|  | USA | P1640L |  |
|  | Australia | T1653I |  |
|  | Scotland | P1692L |  |
|  | France | A1698S |  |
|  | USA | M1769I |  |
|  | USA | P1786L |  |
|  | Wuhan | V1798I |  |
|  | Iceland | P1811S |  |
|  | USA | K1817N |  |
|  | Belgium | T1822I |  |
|  | Iceland | T1822I |  |
|  | USA | T1840I |  |
|  | Japan | K1860N |  |
|  | USA | K1860N |  |
|  | Iceland | S1872N |  |
|  | Shanghai | P1921S |  |
|  | USA | S1924G |  |
|  | Netherlands | A1997V |  |
|  | South_Korea | A1997V |  |
|  | Iceland | A2001T |  |
|  | USA | T2007I |  |
|  | Malaysia | S2015R |  |
|  | Australia | T2016K |  |
|  | Malaysia | T2016K |  |
|  | Saudi_Arabia | T2016K |  |
|  | USA | T2016K |  |
|  | Iceland | P2018S |  |
|  | USA | P2018S |  |
|  | Shanghai | A2033V |  |
|  | USA | E2053D |  |
|  | Netherlands | P2055S |  |
|  | England | E2070G |  |
|  | China | P2079L |  |
|  | Guangdong | P2079L |  |
|  | India | P2079L |  |
|  | Shanghai | S2083I |  |
|  | USA | T2124I |  |
|  | USA | K2143R |  |
|  | India | P2144S |  |
|  | USA | P2144L |  |
|  | USA | T2183I |  |
|  | China | S2185I |  |
|  | Netherlands | N2187T |  |
|  | Shenzhen | M2194T |  |
|  | Switzerland | A2199V |  |
|  | Italy | I2231L |  |
|  | Wuhan | L2235I |  |
|  | England | S2237I |  |
|  | England | V2238I |  |
|  | Wales | S2242F |  |
|  | Wuhan | I2244T |  |
|  | Australia | A2249V |  |
|  | Iceland | A2249V |  |
|  | Netherlands | A2249V |  |
|  | USA | A2249V |  |
|  | Wuhan | G2251S |  |
|  | USA | N2272D |  |
|  | Shanghai | S2293R |  |
|  | China | T2300I |  |
|  | Shanghai | A2325V |  |
|  | Shandong | A2345V |  |
|  | Denmark | P2376L |  |
|  | USA | S2517F |  |
|  | Wuhan | G2534V |  |
|  | Wuhan | D2579A |  |
|  | Japan | V2580A |  |
|  | Belgium | N2603D |  |
|  | Iceland | N2603D |  |
|  | USA | M2606I |  |
|  | Japan | S2625F |  |
|  | Jiangxi | C2670Y |  |
|  | Australia | L2688F |  |
|  | Slovakia | Q2702H |  |
|  | Wuhan | N2708S |  |
| nsp4 | Australia | M2796I |  |
|  | Canada | M2796I |  |
|  | Congo | M2796L |  |
|  | France | M2796V |  |
|  | Kuwait | M2796I |  |
|  | USA | I2806V |  |
|  | USA | D2835N |  |
|  | Germany | T2872M |  |
|  | Iceland | L2882F |  |
|  | Shanghai | F2884C |  |
|  | Peru | N2894D |  |
|  | Wuhan | F2908I |  |
|  | Japan | G2959D |  |
|  | Pakistan | P2965L |  |
|  | USA | S2972F |  |
|  | Spain | D2980G |  |
|  | Shanghai | A3023V |  |
|  | Shanghai | I3035M |  |
|  | Belgium | T3058I |  |
|  | France | T3058I |  |
|  | Shanghai | T3058S |  |
|  | Japan | A3070V |  |
|  | USA | A3070V |  |
|  | Brazil | F3071Y |  |
|  | Chile | F3071Y |  |
|  | France | F3071Y |  |
|  | Georgia | F3071Y |  |
|  | Netherlands | F3071Y |  |
|  | Shanghai | F3071Y |  |
|  | Spain | F3071Y |  |
|  | USA | F3071Y |  |
|  | Germany | G3072C |  |
|  | Taiwan | G3072C |  |
|  | Australia | E3073G |  |
|  | Taiwan | H3076Y |  |
|  | Wuhan | T3090I |  |
|  | Shenzhen | S3099L |  |
|  | England | L3123F |  |
|  | USA | C3147L |  |
|  | Australia | Y3160H |  |
|  | Shanghai | A3209S |  |
|  | USA | A3220V |  |
|  | USA | A3229V |  |
|  | Hong_Kong | H3233Y |  |
|  | Australia | T3258I |  |
| nsp5 | Denmark | G3278S |  |
|  | England | G3278S |  |
|  | Iceland | G3278S |  |
|  | Brazil | G3334S |  |
|  | Germany | G3334S |  |
|  | Georgia | L3352F |  |
|  | USA | L3352F |  |
|  | Iceland | K3353R |  |
|  | Shanghai | K3353R |  |
|  | Netherlands | V3420I |  |
|  | USA | A3436V |  |
|  | Iceland | T3459M |  |
|  | USA | L3483F |  |
|  | France | T3488I |  |
|  | USA | Y3500H |  |
|  | USA | A3518V |  |
| nsp6 | USA | V3593F |  |
|  | Australia | L3602F |  |
|  | Australia | L3606F |  |
|  | Belgium | L3606F |  |
|  | Brazil | L3606F |  |
|  | Canada | L3606F |  |
|  | Chongqing | L3606F |  |
|  | Congo | L3606F |  |
|  | England | L3606F |  |
|  | Finland | L3606F |  |
|  | France | L3606F |  |
|  | Georgia | L3606F |  |
|  | Germany | L3606F |  |
|  | Hangzhou | L3606F |  |
|  | Hong_Kong | L3606F |  |
|  | Iceland | L3606F |  |
|  | Italy | L3606F |  |
|  | Japan | L3606F |  |
|  | Kuwait | L3606F |  |
|  | Luxembourg | L3606F |  |
|  | Malaysia | L3606F |  |
|  | Netherlands | L3606F |  |
|  | Norway | L3606F |  |
|  | Pakistan | L3606F |  |
|  | Portugal | L3606F |  |
|  | Saudi_Arabia | L3606F |  |
|  | Scotland | L3606F |  |
|  | Shandong | L3606F |  |
|  | Shanghai | L3606F |  |
|  | Singapore | L3606F |  |
|  | South_Korea | L3606F |  |
|  | Spain | L3606F |  |
|  | Switzerland | L3606F |  |
|  | Taiwan | L3606F |  |
|  | USA | L3606F |  |
|  | Wales | L3606F |  |
|  | Yunnan | L3606F |  |
|  | Netherlands | A3615V |  |
|  | Japan | C3682F |  |
|  | Iceland | A3686V |  |
|  | Belgium | T3716I |  |
|  | China | Y3722C |  |
|  | Shanghai | Y3722C |  |
|  | Netherlands | N3725D |  |
|  | Portugal | L3754F |  |
|  | USA | I3758T |  |
|  | Japan | E3764D |  |
|  | Australia | C3790F |  |
|  | Spain | T3791S |  |
|  | USA | L3829F |  |
|  | Wuhan | N3833K |  |
| nsp7 | NanChang | S3884L |  |
|  | France | Q3890H |  |
| nsp8 | USA | S3949N |  |
|  | Iceland | A3958V |  |
|  | Wuhan | T3959N |  |
|  | England | A3995V |  |
|  | Wuhan | M4029L |  |
|  | Australia | T4031I |  |
|  | Switzerland | T4031I |  |
| nsp9 | France | S4153P |  |
|  | USA | L4182F |  |
|  | France | G4201R |  |
|  | USA | T4217I |  |
|  | Russia | G4244R |  |
| nsp10 | Netherlands | A4285V |  |
|  | Switzerland | T4304I |  |
|  | Iceland | D4317E |  |
|  | Sweden | F4321L |  |
|  | Netherlands | L4365F |  |
|  | Iceland | T4371N |  |
| nsp12 | Iceland | S4398L |  |
|  | USA | G4416Y |  |
|  | Belgium | T4417I |  |
|  | Jiangsu | G4435V |  |
|  | Singapore | Y4471C |  |
|  | Guangdong | T4476I |  |
|  | Portugal | K4482R |  |
|  | Australia | A4488V |  |
|  | Malaysia | A4488V |  |
|  | Saudi_Arabia | A4488V |  |
|  | USA | A4488V |  |
|  | USA | K4494R |  |
|  | USA | M4501V |  |
|  | Iceland | T4532I |  |
|  | Switzerland | T4532I |  |
|  | USA | D4545G |  |
|  | England | D4552V |  |
|  | Malaysia | G4570S |  |
|  | Australia | G4619C |  |
|  | USA | S4620N |  |
|  | USA | K4654N |  |
|  | England | T4667M |  |
|  | Australia | P4714L |  |
|  | Australia | P4714L |  |
|  | Belgium | P4714L |  |
|  | Brazil | P4714L |  |
|  | Chile | P4714L |  |
|  | Congo | P4714L |  |
|  | Czech_Republic | P4714L |  |
|  | Denmark | P4714L |  |
|  | England | P4714L |  |
|  | Finland | P4714L |  |
|  | France | P4714L |  |
|  | Georgia | P4714L |  |
|  | Germany | P4714L |  |
|  | Hungary | P4714L |  |
|  | Iceland | P4714L |  |
|  | Italy | P4714L |  |
|  | Japan | P4714L |  |
|  | Lithuania | P4714L |  |
|  | Luxembourg | P4714L |  |
|  | Mexico | P4714L |  |
|  | NanChang | P4714L |  |
|  | Netherlands | P4714L |  |
|  | New_Zealand | P4714L |  |
|  | Peru | P4714L |  |
|  | Portugal | P4714L |  |
|  | Russia | P4714L |  |
|  | Saudi_Arabia | P4714L |  |
|  | Scotland | P4714L |  |
|  | Slovakia | P4714L |  |
|  | South_Africa | P4714L |  |
|  | Spain | P4714L |  |
|  | Switzerland | P4714L |  |
|  | Taiwan | P4714L |  |
|  | USA | P4714L |  |
|  | Vietnam | P4714L |  |
|  | Wales | P4714L |  |
|  | Tianmen | P4719S |  |
|  | Tianmen | A4773V |  |
|  | USA | A4791S |  |
|  | Kuwait | V4796I |  |
|  | Netherlands | V4796F |  |
|  | India | A4797V |  |
|  | China | A4834V |  |
|  | Iceland | A4840V |  |
|  | USA | A4840V |  |
|  | USA | I4857V |  |
|  | Iceland | N4880K |  |
|  | South_Korea | A4917V |  |
|  | Georgia | R5031H |  |
|  | Netherlands | A5047V |  |
|  | Congo | M5059I |  |
|  | Netherlands | A5090S |  |
|  | USA | N5125T |  |
|  | USA | G5165S |  |
|  | Iceland | D5195Y |  |
|  | Iceland | T5197I |  |
|  | France | K5198R |  |
|  | USA | H5201L |  |
|  | Japan | G5214S |  |
|  | Iceland | H5263Y |  |
|  | Netherlands | D5270Y |  |
|  | USA | D5270Y |  |
|  | Taiwan | W5307C |  |
| nsp13 | Netherlands | V5372I |  |
|  | USA | V5372I |  |
|  | Netherlands | S5397A |  |
|  | USA | P5400L |  |
|  | China | S5403G |  |
|  | Guangdong | S5423I |  |
|  | Guangzhou | S5423I |  |
|  | USA | V5492F |  |
|  | Hangzhou | D5527Y |  |
|  | Iceland | V5533I |  |
|  | India | T5537I |  |
|  | USA | V5549L |  |
|  | Iceland | P5561L |  |
|  | Shanghai | P5561L |  |
|  | USA | V5570F |  |
|  | USA | T5578I |  |
|  | Beijing | L5579F |  |
|  | USA | Q5598K |  |
|  | Iceland | H5613Y |  |
|  | South_Korea | H5613Y |  |
|  | South_Korea | A5619T |  |
|  | Belgium | I5650V |  |
|  | USA | P5687S |  |
|  | Shanghai | A5702V |  |
|  | USA | R5715C |  |
|  | USA | T5736I |  |
|  | Iceland | I5755T |  |
|  | Portugal | S5791L |  |
|  | Spain | S5791L |  |
|  | USA | S5791L |  |
|  | Belgium | S5808L |  |
|  | Singapore | V5819A |  |
|  | Australia | P5827L |  |
|  | Iceland | P5827L |  |
|  | USA | P5827L |  |
|  | England | A5832V |  |
|  | Australia | Y5864C |  |
|  | Canada | Y5864C |  |
|  | Iceland | Y5864C |  |
|  | USA | Y5864C |  |
|  | USA | T5873A |  |
|  | Netherlands | A5921V |  |
| nsp14 | USA | S5952G |  |
|  | USA | G5961V |  |
|  | Iceland | G5983C |  |
|  | France | R6000L |  |
|  | USA | P6045L |  |
|  | Congo | I6074T |  |
|  | England | I6074T |  |
|  | Guangdong | L6101F |  |
|  | Guangzhou | L6101F |  |
|  | Denmark | T6130I |  |
|  | Japan | T6130I |  |
|  | Netherlands | C6132F |  |
|  | France | T6139A |  |
|  | China | C6140F |  |
|  | USA | F6141Y |  |
|  | New_Zealand | F6157L |  |
|  | USA | F6157L |  |
|  | Chile | V6214F |  |
|  | Australia | P6221S |  |
|  | France | P6221S |  |
|  | NanChang | A6244V |  |
|  | USA | L6290F |  |
|  | USA | D6303A |  |
|  | Sichuan | F6308Y |  |
|  | Portugal | P6336S |  |
|  | Iceland | P6375L |  |
|  | Canada | A6406V |  |
|  | Belgium | L6417F |  |
|  | Shanghai | L6417F |  |
|  | NanChang | D6420N |  |
|  | USA | M6425I |  |
|  | Taiwan | S6427L |  |
|  | China | T6448I |  |
| nsp15 | Congo | S6452C |  |
|  | USA | N6455K |  |
|  | USA | V6460F |  |
|  | France | V6473L |  |
|  | New_Zealand | V6473L |  |
|  | USA | V6473L |  |
|  | Guangdong | N6480D |  |
|  | Iceland | T6484I |  |
|  | Australia | A6532V |  |
|  | Hangzhou | K6560E |  |
|  | Shenzhen | E6564D |  |
|  | USA | T6565M |  |
|  | Australia | I6594V |  |
|  | Netherlands | T6595I |  |
|  | Kuwait | S6598C |  |
|  | USA | E6621K |  |
|  | Belgium | A6622V |  |
|  | France | V6623I |  |
|  | USA | D6634Y |  |
|  | France | P6656L |  |
|  | England | A6668V |  |
|  | Wales | A6668V |  |
|  | Iceland | D6670N |  |
|  | Netherlands | D6670N |  |
|  | USA | F6672L |  |
|  | Iceland | E6674G |  |
|  | Shanghai | D6690N |  |
|  | Shanghai | A6706S |  |
|  | Portugal | E6711K |  |
|  | Switzerland | E6711K |  |
|  | Congo | S6712L |  |
|  | Iceland | D6718Y |  |
|  | England | D6723Y |  |
|  | Shanghai | V6754F |  |
|  | USA | S6763F |  |
| nsp16 | Shanghai | P6809S |  |
|  | Japan | M6814I |  |
|  | Japan | T6832I |  |
|  | France | G6874R |  |
|  | South_Korea | T6890M |  |
|  | Belgium | G6910C |  |
|  | Australia | L6923F |  |
|  | Wuhan | K6957R |  |
|  | Vietnam | A6959S |  |
|  | Wuhan | D7017N |  |
|  | Spain | R7029G |  |
|  | USA | S7038F |  |
|  | USA | S7040F |  |

| **S glycoprotein** | | |
| --- | --- | --- |
|  |  |  |
|  |  |  |
| **Country** | **Amino acid Change(ref:position:strain)** | **Total Nucleotide mutations** |
| England | L5F | 183 |
| Iceland | L5F |  |
| Japan | L5F |  |
| USA | L5F |  |
| Canada | L8V |  |
| canine | L8V |  |
| Hong_Kong | L8V |  |
| Iceland | L18F |  |
| Henan | Y28D |  |
| Wuhan | F32I |  |
| Wuhan | F32V |  |
| Guangdong | H49Y |  |
| Jiangsu | H49Y |  |
| Shanghai | H49Y |  |
| USA | H49Y |  |
| France | L54F |  |
| USA | L54F |  |
| Malaysia | H69D |  |
| Malaysia | S71T |  |
| USA | S71F |  |
| Malaysia | G72W |  |
| Malaysia | T73F |  |
| Malaysia | N74H |  |
| England | T76I |  |
| Shanghai | S98F |  |
| Japan | I119V |  |
| France | V120I |  |
| Hong_Kong | D138Y |  |
| Iceland | Y145H |  |
| USA | H146Y |  |
| Spain | N148K |  |
| Spain | N149H |  |
| China | M153T |  |
| USA | F157L |  |
| Iceland | M177I |  |
| Japan | G181V |  |
| USA | G181V |  |
| Switzerland | K202N |  |
| France | V213L |  |
| South_Korea | S221W |  |
| Finland | Q239K |  |
| Netherlands | Q239K |  |
| Australia | S247R |  |
| Hangzhou | S247R |  |
| Belgium | S254F |  |
| Germany | S254F |  |
| Netherlands | S254F |  |
| USA | W258L |  |
| USA | Q321L |  |
| Wales | V341I |  |
| USA | A348T |  |
| Shenzhen | N354D |  |
| Shenzhen | D364Y |  |
| France | V367F |  |
| Hong_Kong | V367F |  |
| Shanghai | K378R |  |
| Guangdong | Q409E |  |
| Finland | A435S |  |
| Belgium | K458R |  |
| USA | G476S |  |
| USA | V483A |  |
| France | Y508H |  |
| Belgium | H519P |  |
| USA | T547I |  |
| Shanghai | K558N |  |
| Denmark | L611F |  |
| Belgium | D614G |  |
| Belgium | D614G |  |
| Brazil | D614G |  |
| Australia | D614G |  |
| Chile | D614G |  |
| Congo | D614G |  |
| Czech_Republic | D614G |  |
| Denmark | D614G |  |
| England | D614G |  |
| Finland | D614G |  |
| France | D614G |  |
| Georgia | D614G |  |
| Germany | D614G |  |
| Hungary | D614G |  |
| Iceland | D614G |  |
| Ireland | D614G |  |
| Italy | D614G |  |
| Japan | D614G |  |
| Lithuania | D614G |  |
| Luxembourg | D614G |  |
| Mexico | D614G |  |
| Netherlands | D614G |  |
| NetherlandsL | D614G |  |
| New_Zealand | D614G |  |
| Norway | D614G |  |
| Panama | D614G |  |
| Peru | D614G |  |
| Portugal | D614G |  |
| Russia | D614G |  |
| Saudi_Arabia | D614G |  |
| Scotland | D614G |  |
| Shanghai | D614G |  |
| Slovakia | D614G |  |
| South_Africa | D614G |  |
| Spain | D614G |  |
| Switzerland | D614G |  |
| Taiwan | D614G |  |
| USA | D614G |  |
| Vietnam | D614G |  |
| Wales | D614G |  |
| Wuhan | D614G |  |
| Tianmen | V615L |  |
| France | A653V |  |
| Georgia | E654Q |  |
| USA | H655Y |  |
| Denmark | Q675H |  |
| Iceland | Q675H |  |
| Norway | Q675H |  |
| Scotland | Q675H |  |
| Hangzhou | R682Q |  |
| Shanghai | N703S |  |
| Belgium | A706V |  |
| Hong_Kong | T719A |  |
| France | I720V |  |
| Wuhan | L752F |  |
| Taiwan | R765L |  |
| Netherlands | V772L |  |
| Germany | E780Q |  |
| Taiwan | T791I |  |
| Sweden | F797C |  |
| Australia | P809S |  |
| USA | P809S |  |
| Netherlands | P812S |  |
| Singapore | F817L |  |
| Congo | L821I |  |
| Iceland | A831V |  |
| Iceland | I834T |  |
| England | D839Y |  |
| Georgia | D839Y |  |
| Iceland | D839Y |  |
| Netherlands | D839E |  |
| Portugal | D839Y |  |
| Portugal | A845S |  |
| Netherlands | A852V |  |
| Beijing | V860Q |  |
| Beijing | L861K |  |
| Shanghai | E868K |  |
| Australia | G889S |  |
| USA | S929I |  |
| India | A930V |  |
| USA | D936Y |  |
| France | S939F |  |
| Iceland | S939F |  |
| Switzerland | S939F |  |
| USA | S939F |  |
| Belgium | S940T |  |
| Australia | S940F |  |
| Belgium | T941A |  |
| Belgium | S943P |  |
| Belgium | S943T |  |
| Shanghai | L948R |  |
| Shanghai | V952F |  |
| Spain | Q954K |  |
| Beijing | F970S |  |
| China | V1040F |  |
| Japan | P1079T |  |
| Japan | K1086R |  |
| Congo | V1122L |  |
| Shenzhen | V1129L |  |
| France | I1132V |  |
| Australia | P1143L |  |
| France | P1143L |  |
| China | D1146E |  |
| France | P1162A |  |
| Shanghai | P1162L |  |
| England | D1165G |  |
| Henan | S1170Y |  |
| Iceland | S1170P |  |
| Ecuador | E1207V |  |
| Netherlands | Y1209H |  |
| Shanghai | I1216T |  |
| Japan | V1228L |  |
| Iceland | M1229I |  |
| Netherlands | M1237I |  |
| Portugal | M1237I |  |
| USA | M1237I |  |
| USA | C1247F |  |
| Wales | C1247F |  |
| Australia | C1254F |  |
| Shenzhen | E1262G |  |
| England | P1263L |  |
| Henan | P1263L |  |
| Iceland | P1263L |  |
| Spain | L1265P |  |
| Netherlands | T1273I |  |

| **Membrane (M) Protein** | | |
| --- | --- | --- |
|  |  |  |
|  |  |  |
| **Country** | **Amino acid Change(ref:position:strain)** | **Total Nucleotide mutations** |
| Switzerland | D3G | 34 |
| Lithuania | D3G |  |
| Belgium | D3G |  |
| Portugal | D3G |  |
| Scotland | D3G |  |
| Iceland | D3G |  |
| Slovakia | D3G |  |
| England | D3G |  |
| Finland | D3G |  |
| Italy | D3G |  |
| Congo | D3G |  |
| USA | D3G |  |
| Shanghai | S4F |  |
| Turkey | V10A |  |
| Wales | L16F |  |
| USA | G25V |  |
| Iceland | C33F |  |
| South_Korea | A40S |  |
| South_Korea | A40S |  |
| South_Korea | I52T |  |
| USA | V70I |  |
| Shenzhen | G78A |  |
| Shanghai | T130P |  |
| England | R158C |  |
| Ireland | T175M |  |
| Belgium | T175M |  |
| Portugal | T175M |  |
| England | T175M |  |
| Iceland | T175M |  |
| Germany | T175M |  |
| Scotland | T175M |  |
| Brazil | T175M |  |
| Finland | T175M |  |
| Switzerland | T175M |  |
| France | T175M |  |
| Netherlands | T175M |  |
| USA | T175M |  |
| Turkey | A194V |  |
| Singapore | D209H |  |
| Malaysia | D209H |  |

| **Envelop (E) protein** | | |
| --- | --- | --- |
|  |  |  |
|  |  |  |
| **Country** | **Amino acid Change(ref:position:strain)** | **Total Nucleotide mutations** |
| Canada | S6L | 27 |
| Shanghai | T30I |  |
| South_Korea | L37H |  |
| Shanghai | I46F |  |
| England | V58L |  |
| Australia | S68F |  |
| Guangdong | S68C |  |
| Guangzhou | S68C |  |
| Spain | P71L |  |
| Guangzhou | D72Y |  |
| France | D72G |  |
| Iceland | L73F |  |

| **Nucleocapsid (N) Protein** | | |
| --- | --- | --- |
|  |  |  |
|  |  |  |
| **Country** | **Amino acid Change(ref:position:strain)** | **Total Nucleotide mutations** |
| USA | D3Y | 148 |
| USA | N4D |  |
| Netherlands | Q9H |  |
| USA | Q9H |  |
| Australia | P13L |  |
| Malaysia | P13L |  |
| Portugal | P13S |  |
| Saudi_Arabia | P13L |  |
| USA | P13L |  |
| NewZealand | D22G |  |
| Shanghai | S23P |  |
| Iceland | T24N |  |
| Netherlands | T24N |  |
| USA | T24I |  |
| England | Q28H |  |
| USA | A35T |  |
| USA | P46S |  |
| Australia | E62K |  |
| Chile | D103Y |  |
| Iceland | D103Y |  |
| Wuhan | W108C |  |
| Iceland | P117S |  |
| Iceland | A119V |  |
| Netherlands | A119V |  |
| Shandong | L121H |  |
| Portugal | P122L |  |
| Spain | P122L |  |
| USA | P122L |  |
| Iceland | W132Y |  |
| Iceland | A134V |  |
| Shanghai | H145Y |  |
| USA | I146F |  |
| Shenzhen | T148I |  |
| Wales | P151L |  |
| USA | A152S |  |
| England | A156S |  |
| Netherlands | A156S |  |
| Iceland | P168S |  |
| USA | S183Y |  |
| Belgium | R185C |  |
| Shanghai | R185C |  |
| USA | R185C |  |
| Shanghai | S186Y |  |
| Iceland | S188L |  |
| Netherlands | S188P |  |
| Switzerland | S188L |  |
| Taiwan | S188P |  |
| Guangdong | S190G |  |
| Belgium | R191L |  |
| Shanghai | R191L |  |
| Shanghai | S193I |  |
| Wales | S193I |  |
| Wales | S193I |  |
| Canada | S194L |  |
| China | S194L |  |
| Foshan | S194L |  |
| Iceland | S194L |  |
| Shanghai | S194L |  |
| Shenzhen | S194L |  |
| USA | S194L |  |
| Wales | S194L |  |
| Belgium | R195K |  |
| France | R195K |  |
| Brazil | S197L |  |
| Chile | S197L |  |
| France | S197L |  |
| Georgia | S197L |  |
| Netherlands | S197L |  |
| Portugal | S197L |  |
| Spain | S197L |  |
| USA | S197L |  |
| Wales | S197L |  |
| Singapore | P199S |  |
| USA | G200S |  |
| Australia | S202N |  |
| China | S202N |  |
| Guangdong | S202N |  |
| Netherlands | S202N |  |
| Shanghai | S202N |  |
| Singapore | S202N |  |
| USA | S202N |  |
| Australia | R203K |  |
| Belgium | R203K |  |
| Brazil | R203K |  |
| Chile | R203K |  |
| Czech_Republic | R203K |  |
| Denmark | R203K |  |
| England | R203K |  |
| Finland | R203K |  |
| France | R203K |  |
| Germany | R203K |  |
| Hungary | R203K |  |
| Iceland | R203K |  |
| Ireland | R203K |  |
| Italy | R203K |  |
| Mexico | R203K |  |
| Netherlands | R203K |  |
| New_Zealand | R203K |  |
| Nigeria | R203K |  |
| Peru | R203K |  |
| Portugal | R203K |  |
| Scotland | R203K |  |
| Spain | R203K |  |
| Switzerland | R203K |  |
| Taiwan | R203K |  |
| USA | R203K |  |
| Vietnam | R203K |  |
| Wales | R203K |  |
| Australia | G204R |  |
| Belgium | G204R |  |
| Brazil | G204R |  |
| Chile | G204R |  |
| Czech_Republic | G204R |  |
| Denmark | G204R |  |
| England | G204R |  |
| Finland | G204R |  |
| France | G204R |  |
| Germany | G204R |  |
| Hungary | G204R |  |
| Iceland | G204R |  |
| Ireland | G204R |  |
| Italy | G204R |  |
| Mexico | G204R |  |
| Netherlands | G204R |  |
| New_Zealand | G204R |  |
| Nigeria | G204R |  |
| Peru | G204R |  |
| Portugal | G204R |  |
| Scotland | G204R |  |
| Spain | G204R |  |
| Switzerland | G204R |  |
| Taiwan | G204R |  |
| USA | G204R |  |
| Vietnam | G204R |  |
| Wales | G204R |  |
| Australia | T205I |  |
| Shanghai | T205I |  |
| USA | N213Y |  |
| Portugal | A217V |  |
| USA | S235P |  |
| Colombia | G238C |  |
| Italy | V246I |  |
| Wuhan | K249I |  |
| USA | A252S |  |
| Iceland | Q289H |  |
| Czech_Republic | I292T |  |
| Netherlands | I292T |  |
| Switzerland | I292T |  |
| Iceland | A308S |  |
| USA | G321D |  |
| Hong_Kong | M322I |  |
| USA | P326L |  |
| USA | S327L |  |
| England | G328V |  |
| Iceland | T334I |  |
| Netherlands | A336V |  |
| Netherlands | L339F |  |
| Beijing | D343V |  |
| China | P344S |  |
| Guangdong | P344S |  |
| Guangzhou | P344S |  |
| Japan | P344S |  |
| Shanghai | P344S |  |
| England | D348Y |  |
| Iceland | V350F |  |
| Japan | I351V |  |
| Iceland | F363S |  |
| Hong_Kong | P365S |  |
| Hong_Kong | P368S |  |
| Netherlands | K370N |  |
| England | K373N |  |
| Netherlands | K373N |  |
| NewZealand | D377G |  |
| Finland | E378Q |  |
| Iceland | E378Q |  |
| USA | T391I |  |
| Japan | A397V |  |
| USA | A397S |  |
| Wales | A397S |  |
| Netherlands | D402Y |  |
| Guangdong | S404A |  |
| Shanghai | S404A |  |
| Iceland | A414S |  |
| Iceland | Q418H |  |

| **ORF3a** | | |
| --- | --- | --- |
|  |  |  |
|  |  |  |
| **Country** | **Amino acid Change(Refpositionstrain)** | **Total Nucleotide mutations** |
| Netherlands | F8L | 92 |
| Iceland | G11V |  |
| England | V13L |  |
| Iceland | V13L |  |
| USA | V13L |  |
| Wales | V13L |  |
| France | T14I |  |
| Australia | A31T |  |
| Hungary | A31T |  |
| Wales | T34M |  |
| Portugal | G49C |  |
| France | A54V |  |
| Australia | Q57H |  |
| Belgium | Q57H |  |
| Congo | Q57H |  |
| Denmark | Q57H |  |
| Finland | Q57H |  |
| France | Q57H |  |
| Georgia | Q57H |  |
| Germany | Q57H |  |
| Iceland | Q57H |  |
| Luxembourg | Q57H |  |
| Netherlands | Q57H |  |
| Portugal | Q57H |  |
| Russia | Q57H |  |
| Saudi_Arabia | Q57H |  |
| Slovakia | Q57H |  |
| USA | Q57H |  |
| Wales | Q57H |  |
| Singapore | A59D |  |
| Netherlands | K61N |  |
| Wales | I62V |  |
| Iceland | K67N |  |
| Netherlands | S74A |  |
| Australia | K75E |  |
| Saudi_Arabia | K75E |  |
| New_Zealand | G76S |  |
| Cambodia | V88L |  |
| USA | V88A |  |
| Japan | T89I |  |
| Henan | H93Q |  |
| Wales | H93Y |  |
| Belgium | A99V |  |
| Iceland | A99V |  |
| Luxembourg | A99V |  |
| Shanghai | M125I |  |
| South_Korea | W128L |  |
| Japan | L140V |  |
| Netherlands | D155Y |  |
| Guangdong | S166L |  |
| Shanghai | S171L |  |
| Wales | G172C |  |
| Guangdong | T175K |  |
| Portugal | T175I |  |
| Jiangsu | T176I |  |
| USA | Y184H |  |
| Iceland | Y189C |  |
| Taiwan | E191G |  |
| Brazil | G196V |  |
| Chile | G196V |  |
| France | G196V |  |
| Georgia | G196V |  |
| Netherlands | G196V |  |
| Portugal | G196V |  |
| Spain | G196V |  |
| USA | G196V |  |
| Japan | G224C |  |
| USA | G224V |  |
| Shenzhen | E239V |  |
| Shenzhen | D250V |  |
| Australia | G251V |  |
| Belgium | G251V |  |
| Brazil | G251V |  |
| Canada | G251V |  |
| Congo | G251V |  |
| England | G251V |  |
| Finland | G251V |  |
| France | G251V |  |
| Georgia | G251V |  |
| Guangdong | G251V |  |
| Hangzhou | G251V |  |
| Hong_Kong | G251V |  |
| Iceland | G251V |  |
| Italy | G251V |  |
| Luxembourg | G251V |  |
| Netherlands | G251V |  |
| NewZealand | G251V |  |
| Norway | G251V |  |
| Portugal | G251V |  |
| Scotland | G251V |  |
| Shanghai | G251V |  |
| Singapore | G251V |  |
| South_Korea | G251V |  |
| Spain | G251V |  |
| Sweden | G251V |  |
| Switzerland | G251V |  |
| Taiwan | G251V |  |
| USA | G251V |  |
| USA | G251C |  |
| Wales | G251V |  |
| Portugal | S253F |  |
| USA | G254R |  |
| South_Korea | V259L |  |
| China | T269M |  |
| Hong_Kong | T269M |  |
| Iceland | V273L |  |
| Iceland | L275F |  |

| **ORF 6** | | |
| --- | --- | --- |
|  |  |  |
|  |  |  |
| **Country** | **Amino acid Change(Refpositionstrain)** | **Total Nucleotide mutations** |
| France | H3Y | 8 |
| Japan | V5F |  |
| USA | V9F |  |
| USA | K42N |  |
| Wales | E55D |  |

| **ORF7a** | | |
| --- | --- | --- |
|  |  |  |
|  |  |  |
| **Country** | **Amino acid Change(ref:position:strain)** | **Total Nucleotide mutations** |
| Iceland | L5P | 46 |
| USA | A13V |  |
| Iceland | A13T |  |
| Wales | A13V |  |
| Wales | G26R |  |
| Iceland | T28I |  |
| Iceland | V29L |  |
| Wuhan | P34S |  |
| Portugal | G38V |  |
| Iceland | P45L |  |
| Iceland | H47Y |  |
| Shanghai | F54S |  |
| Iceland | T61I |  |
| Shandong | H73Q |  |
| Kuwait | V74F |  |
| England | S81L |  |
| New_Zealand | S81L |  |
| USA | S81L |  |
| Shanghai | Q90P |  |
| Shanghai | E92D |  |
| Shanghai | V93D |  |
| Shanghai | I100F |  |
| USA | I110T |  |
| Spain | L116P |  |
| Scotland | T120I |  |

| **ORF7b** | | |
| --- | --- | --- |
|  |  |  |
|  |  |  |
| **Country** | **Amino acid Change(ref:position:strain)** | **Total Nucleotide mutations** |
| Germany | L32F | 8 |
| Portugal | A15S |  |
| USA | C41F |  |
|  |  |  |

| **ORF8** | | |
| --- | --- | --- |
|  |  |  |
|  |  |  |
| **Country** | **Amino acid Change(ref:position:strain)** | **Total Nucleotide mutations** |
| USA | T11I | 33 |
| USA | S24L |  |
| USA | P36S |  |
| Beijing | V62L |  |
| Guangdong | V62L |  |
| Hong_Kong | V62L |  |
| New_Zealand | V62L |  |
| Shanghai | V62L |  |
| Sichuan | V62L |  |
| USA | V62L |  |
| Vietnam | V62L |  |
| Wales | V62L |  |
| USA | A65S |  |
| Jingzhou | S67F |  |
| USA | S69L |  |
| Turkey | Q72H |  |
| Netherlands | Y73H |  |
| Guangdong | G77C |  |
| Anhui | L84S |  |
| Australia | L84S |  |
| Beijing | L84S |  |
| Belgium | L84S |  |
| Brazil | L84S |  |
| Canada | L84S |  |
| Chile | L84S |  |
| China | L84S |  |
| Chongqing | L84S |  |
| Colombia | L84S |  |
| England | L84S |  |
| France | L84S |  |
| Fujian | L84S |  |
| Georgia | L84S |  |
| Germany | L84S |  |
| Guangdong | L84S |  |
| Guangzhou | L84S |  |
| Hangzhou | L84S |  |
| Henan | L84S |  |
| Hong_Kong | L84S |  |
| Iceland | L84S |  |
| India | L84S |  |
| Japan | L84S |  |
| Malaysia | L84S |  |
| Netherlands | L84S |  |
| New_Zealand | L84S |  |
| Portugal | L84S |  |
| Shandong | L84S |  |
| Shanghai | L84S |  |
| Shenzhen | L84S |  |
| Sichuan | L84S |  |
| Singapore | L84S |  |
| South_Korea | L84S |  |
| Spain | L84S |  |
| Taiwan | L84S |  |
| Tianmen | L84S |  |
| USA | L84S |  |
| Vietnam | L84S |  |
| Wales | L84S |  |
| Wuhan | L84S |  |
| Yunnan | L84S |  |
| Henan | P85S |  |
| Guangdong | L95F |  |
| Jingzhou | S97N |  |
| USA | H112Q |  |
| Shanghai | V114I |  |

| **ORF10** | | |
| --- | --- | --- |
|  |  |  |
|  |  |  |
| **Country** | **Amino acid Change(ref:position:strain)** | **Total Nucleotide mutations** |
| USA | A8V | 17 |
| Finland | P10S |  |
| Japan | T12M |  |
| England | I13M |  |
| Belgium | Y14C |  |
| Australia | S23F |  |
| Australia | R24C |  |
| USA | R24L |  |
| USA | A28V |  |
| Shanghai | T38I |  |

| **5' UTR** | |
| --- | --- |
|  |  |
|  |  |
|  |  |
| Total nucleotide mutations | 105 |
|  |  |

| 3' UTR | |
| --- | --- |
|  |  |
|  |  |
|  |  |
| Total nucleotide mutations | 158 |
|  |  |

| Spacer region | |
| --- | --- |
|  |  |
|  |  |
|  |  |
| Total nucleotide mutations | 6 |
|  |  |

**Supplementary Table 1c: Deletion analysis results**

| **Position** | | **Strains** | **Deleted Amino acid position in polyprotein** | **Deleted Amino acid position in ORF** | **Deleted amino acid Name** |
| --- | --- | --- | --- | --- | --- |
| nsp1 (94-117) | Japan/AI/I-004/2020\|EPI_ISL_407084\|2020-01-25 | | 32-39 | 32-39 |  |
| nsp1 (243-257) | USA/CA6/2020\|EPI_ISL_410044\|2020-01-27 | | 82-86 | 82-86 | GHVMV |
|  | Japan/DP0058/2020\|EPI_ISL_416568\|2020-02-15 | | 82-86 | 82-86 | GHVMV |
|  | Japan/DP0286/2020\|EPI_ISL_416588\|2020-02-16 | | 82-86 | 82-86 | GHVMV |
|  | Netherlands/NoordBrabant_31/2020\|EPI_ISL_414540\|2020-03-08 | | 82-86 | 82-86 | GHVMV |
|  | USA/CruiseA-18/2020\|EPI_ISL_413623\|2020-02-24 | | 82-86 | 82-86 | GHVMV |
| nsp1 (253-255) | USA/WA-UW90/2020\|EPI_ISL_416446\|2020-03-10 | | 85 | 85 | M |
|  | Netherlands/NoordBrabant_54/2020\|EPI_ISL_415510\|2020-03-09 | | 85 | 85 | M |
| nsp1 (251-253) | USA/UT-00031/2020\|EPI_ISL_417973\|2020-03-24 | | 85 | 85 | M |
|  | USA/WA-UW53/2020\|EPI_ISL_415618\|2020-03-09 | | 85 | 85 | M |
| nsp1 (404-406) | USA/WA-UW61/2020\|EPI_ISL_415626\|2020-03-10 | | 136 | 136 | Y |
|  | USA/WA-UW151/2020\|EPI_ISL_416689\|2020-03-14 | | 136 | 136 | Y |
| nsp1 (421-429) | England/20106005303/2020\|EPI_ISL_417243\|2020-03-03 | | 141-143 | 141-143 | KSF |
|  | Canada/ON-VIDO-01/2020\|EPI_ISL_413015\|2020-01-23 | | 141-143 | 141-143 | KSF |
|  | Iceland/147/2020\|EPI_ISL_417797\|2020-03-13 | | 141-143 | 141-143 | KSF |
|  | USA/CA6/2020\|EPI_ISL_410044\|2020-01-27 | | 141-143 | 141-143 | KSF |
|  | USA/WA-UW256/2020\|EPI_ISL_418041\|2020-03-14 | | 141-143 | 141-143 | KSF |
| nsp2 (1340-13420) | Netherlands/NoordBrabant_54/2020\|EPI_ISL_415510\|2020-03-09 | | 448 | 268 | D |
|  | Netherlands/NoordBrabant_31/2020\|EPI_ISL_414540\|2020-03-08 | | 448 | 268 | D |
|  | England/20109098906/2020\|EPI_ISL_417296\|2020-03-06 | | 448 | 268 | D |
|  | Portugal/PT0015/2020\|EPI_ISL_418000\|2020-03-10 | | 448 | 268 | D |
|  | Portugal/PT0041/2020\|EPI_ISL_418026\|2020-03-17 | | 448 | 268 | D |
|  | England/200641094/2020\|EPI_ISL_414040\|2020-02-05 | | 448 | 268 | D |
|  | England/200690300/2020\|EPI_ISL_414042\|2020-02-08 | | 448 | 268 | D |
|  | England/200690245/2020\|EPI_ISL_414041\|2020-02-08 | | 448 | 268 | D |
|  | England/200690306/2020\|EPI_ISL_414043\|2020-02-07 | | 448 | 268 | D |
|  | England/20109050106/2020\|EPI_ISL_417263\|2020-03-06 | | 448 | 268 | D |
|  | England/200690756/2020\|EPI_ISL_414044\|2020-02-08 | | 448 | 268 | D |
|  | England/20110097506/2020\|EPI_ISL_417313\|2020-03-08 | | 448 | 268 | D |
|  | England/20109058906/2020\|EPI_ISL_417283\|2020-03-06 | | 448 | 268 | D |
|  | Slovakia/SK-BMC6/2020\|EPI_ISL_417880\|2020-03-08 | | 448 | 268 | D |
|  | England/20110015106/2020\|EPI_ISL_417306\|2020-03-08 | | 448 | 268 | D |
|  | England/20110059306/2020\|EPI_ISL_417312\|2020-03-08 | | 448 | 268 | D |
|  | England/20109050306/2020\|EPI_ISL_417264\|2020-03-04 | | 448 | 268 | D |
|  | England/20109052506/2020\|EPI_ISL_417276\|2020-03-07 | | 448 | 268 | D |
|  | England/20109093706/2020\|EPI_ISL_417286\|2020-03-05 | | 448 | 268 | D |
|  | Iceland/298/2020\|EPI_ISL_417627\|2020-03-18 | | 448 | 268 | D |
|  | Iceland/320/2020\|EPI_ISL_417649\|2020-03-18 | | 448 | 268 | D |
|  | Iceland/299/2020\|EPI_ISL_417628\|2020-03-18 | | 448 | 268 | D |
|  | Iceland/276/2020\|EPI_ISL_417604\|2020-03-17 | | 448 | 268 | D |
|  | Iceland/337/2020\|EPI_ISL_417666\|2020-03-15 | | 448 | 268 | D |
|  | England/20109093606/2020\|EPI_ISL_417285\|2020-03-06 | | 448 | 268 | D |
|  | Wales/PHWC-23A81/2020\|EPI_ISL_418084\|2020-03-12 | | 448 | 268 | D |
|  | Wales/PHWC-2414F/2020\|EPI_ISL_418148\|2020-03-16 | | 448 | 268 | D |
|  | Wales/PHWC-23CB8/2020\|EPI_ISL_418101\|2020-03-15 | | 448 | 268 | D |
|  | Wales/PHWC-23CF4/2020\|EPI_ISL_418105\|2020-03-15 | | 448 | 268 | D |
|  | England/20102000906/2020\|EPI_ISL_415148\|2020-03-03 | | 448 | 268 | D |
|  | Netherlands/Oisterwijk_1364072/2020\|EPI_ISL_413580\|2020-03-02 | | 448 | 268 | D |
|  | Netherlands/Utrecht_1364066/2020\|EPI_ISL_413590\|2020-03-02 | | 448 | 268 | D |
|  | Netherlands/Rotterdam_1363790/2020\|EPI_ISL_413582\|2020-03-01 | | 448 | 268 | D |
|  | Netherlands/Utrecht_12/2020\|EPI_ISL_414462\|2020-03-04 | | 448 | 268 | D |
|  | Netherlands/NoordBrabant_10/2020\|EPI_ISL_414431\|2020-03-02 | | 448 | 268 | D |
|  | Netherlands/Rotterdam_1364040/2020\|EPI_ISL_413583\|2020-03-02 | | 448 | 268 | D |
|  | Netherlands/Naarden_1364774/2020\|EPI_ISL_413577\|2020-03-02 | | 448 | 268 | D |
|  | Netherlands/Andel_1365066/2020\|EPI_ISL_413564\|2020-03-01 | | 448 | 268 | D |
|  | Netherlands/NA_32/2020\|EPI_ISL_415489\|2020-03-13 | | 448 | 268 | D |
|  | Netherlands/NoordBrabant_51/2020\|EPI_ISL_415507\|2020 | | 448 | 268 | D |
|  | Netherlands/NoordBrabant_48/2020\|EPI_ISL_415505\|2020-03-11 | | 448 | 268 | D |
|  | Netherlands/NoordBrabant_11/2020\|EPI_ISL_414432\|2020-03-02 | | 448 | 268 | D |
|  | Netherlands/Hardinxveld_Giessendam_1364806/2020\|EPI_ISL_413573\|2020-03-02 | | 448 | 268 | D |
|  | Netherlands/NoordBrabant_61/2020\|EPI_ISL_415517\|2020 | | 448 | 268 | D |
|  | Netherlands/NoordBrabant_63/2020\|EPI_ISL_415519\|2020 | | 448 | 268 | D |
|  | Netherlands/Tilburg_/2020\|EPI_ISL_413585\|2020 | | 448 | 268 | D |
|  | Netherlands/Loon_op_zand_1363512/2020\|EPI_ISL_413576\|2020-02-29 | | 448 | 268 | D |
|  | Netherlands/NoordBrabant_19/2020\|EPI_ISL_414459\|2020-03-06 | | 448 | 268 | D |
|  | Netherlands/NoordBrabant_18/2020\|EPI_ISL_414458\|2020-03-05 | | 448 | 268 | D |
|  | Netherlands/Coevorden_1363618/2020\|EPI_ISL_413567\|2020 | | 448 | 268 | D |
|  | Netherlands/Dalen_1363624/2020\|EPI_ISL_413568\|2020-03-01 | | 448 | 268 | D |
|  | Netherlands/NoordBrabant_68/2020\|EPI_ISL_415524\|2020-03-12 | | 448 | 268 | D |
|  | Netherlands/NoordBrabant_44/2020\|EPI_ISL_415501\|2020-03-11 | | 448 | 268 | D |
|  | Netherlands/NoordBrabant_57/2020\|EPI_ISL_415513\|2020-03-11 | | 448 | 268 | D |
|  | Netherlands/NoordBrabant_65/2020\|EPI_ISL_415521\|2020 | | 448 | 268 | D |
|  | Netherlands/NoordBrabant_53/2020\|EPI_ISL_415509\|2020 | | 448 | 268 | D |
|  | Netherlands/NoordBrabant_42/2020\|EPI_ISL_415500\|2020-03-10 | | 448 | 268 | D |
|  | Netherlands/Oss_1363500/2020\|EPI_ISL_413581\|2020-02-29 | | 448 | 268 | D |
|  | Netherlands/NA_22/2020\|EPI_ISL_415479\|2020-03-08 | | 448 | 268 | D |
|  | Netherlands/NoordBrabant_12/2020\|EPI_ISL_414452\|2020-03-06 | | 448 | 268 | D |
|  | Netherlands/Tilburg_1363354/2020\|EPI_ISL_413586\|2020-02-27 | | 448 | 268 | D |
|  | Netherlands/NoordBrabant_49/2020\|EPI_ISL_415506\|2020-03-11 | | 448 | 268 | D |
|  | Netherlands/NoordBrabant_52/2020\|EPI_ISL_415508\|2020-03-09 | | 448 | 268 | D |
|  | Netherlands/NoordBrabant_60/2020\|EPI_ISL_415516\|2020-03-11 | | 448 | 268 | D |
|  | Netherlands/Nieuwendijk_1363582/2020\|EPI_ISL_413578\|2020-03-01 | | 448 | 268 | D |
|  | Netherlands/ZuidHolland_7/2020\|EPI_ISL_414467\|2020-03-05 | | 448 | 268 | D |
|  | Netherlands/Overijssel_2/2020\|EPI_ISL_414550\|2020-03-06 | | 448 | 268 | D |
|  | Netherlands/NoordBrabant_34/2020\|EPI_ISL_414543\|2020-03 | | 448 | 268 | D |
|  | Netherlands/Utrecht_12/2020\|EPI_ISL_414551\|2020-03-07 | | 448 | 268 | D |
|  | Netherlands/NoordBrabant_28/2020\|EPI_ISL_414537\|2020-03-08 | | 448 | 268 | D |
|  | Netherlands/NoordBrabant_38/2020\|EPI_ISL_414547\|2020-03-06 | | 448 | 268 | D |
|  | Netherlands/NoordBrabant_24/2020\|EPI_ISL_414533\|2020-03-06 | | 448 | 268 | D |
|  | Netherlands/NoordBrabant_27/2020\|EPI_ISL_414536\|2020-03-08 | | 448 | 268 | D |
|  | Netherlands/NoordBrabant_33/2020\|EPI_ISL_414542\|2020-03 | | 448 | 268 | D |
|  | Netherlands/NoordBrabant_35/2020\|EPI_ISL_414544\|2020-03-09 | | 448 | 268 | D |
|  | Netherlands/NoordBrabant_29/2020\|EPI_ISL_414538\|2020-03-08 | | 448 | 268 | D |
|  | Netherlands/NoordBrabant_30/2020\|EPI_ISL_414539\|2020-03-08 | | 448 | 268 | D |
|  | Netherlands/NoordBrabant_32/2020\|EPI_ISL_414541\|2020-03-08 | | 448 | 268 | D |
|  | Netherlands/ZuidHolland_21/2020\|EPI_ISL_414563\|2020-03-03 | | 448 | 268 | D |
|  | Netherlands/Utrecht_14/2020\|EPI_ISL_414553\|2020-03-09 | | 448 | 268 | D |
|  | Netherlands/NA_5/2020\|EPI_ISL_415494\|2020 | | 448 | 268 | D |
|  | Netherlands/NA_4/2020\|EPI_ISL_415493\|2020 | | 448 | 268 | D |
|  | Netherlands/NA_33/2020\|EPI_ISL_415490\|2020-03-07 | | 448 | 268 | D |
|  | Netherlands/NoordBrabant_66/2020\|EPI_ISL_415522\|2020 | | 448 | 268 | D |
|  | France/RA739/2020\|EPI_ISL_410486\|2020-02-08 | | 448 | 268 | D |
|  | France/Lyon_06573/2020\|EPI_ISL_417335\|2020-03-06 | | 448 | 268 | D |
|  | NewZealand/CoV001/2020\|EPI_ISL_417211\|2020-03-11 | | 448 | 268 | D |
|  | Netherlands/NoordBrabant_47/2020\|EPI_ISL_415504\|2020-03-09 | | 448 | 268 | D |
|  | Wales/PHWC-23D00/2020\|EPI_ISL_418106\|2020-03-13 | | 448 | 268 | D |
|  | England/20106003303/2020\|EPI_ISL_417239\|2020-03-03 | | 448 | 268 | D |
|  | England/20102112102/2020\|EPI_ISL_417218\|2020-03-02 | | 448 | 268 | D |
|  | England/20109093906/2020\|EPI_ISL_417288\|2020-03-05 | | 448 | 268 | D |
|  | Wales/PHWC-240BB/2020\|EPI_ISL_418141\|2020-03-13 | | 448 | 268 | D |
|  | Wales/PHWC-242D3/2020\|EPI_ISL_418164\|2020-03-17 | | 448 | 268 | D |
|  | Wales/PHWC-23B42/2020\|EPI_ISL_418092\|2020-03-12 | | 448 | 268 | D |
|  | England/20108006603/2020\|EPI_ISL_417253\|2020-03-03 | | 448 | 268 | D |
|  | Netherlands/Utrecht_19/2020\|EPI_ISL_415528\|2020-03-12 | | 448 | 268 | D |
|  | Netherlands/Limburg_6/2020\|EPI_ISL_414427\|2020-03-03 | | 448 | 268 | D |
|  | England/20109038906/2020\|EPI_ISL_417261\|2020-03-06 | | 448 | 268 | D |
| nsp8 (12355-12357) | Netherlands/NoordBrabant_49/2020\|EPI_ISL_415506\|2020-03-11 | | 4119 | 177 | S |
|  | Netherlands/NoordBrabant_52/2020\|EPI_ISL_415508\|2020-03-09 | | 4119 | 177 | S |
| nsp15 (20031-20033) | USA/WI1/2020\|EPI_ISL_408670\|2020-01-31 | | 6678 | 277 | L |
| 3-UTR (13-41) | Wuhan/IVDC-HB-envF54/2020\|EPI_ISL_408512 | | N/A | N/A |  |
| 3-UTR (73-84) | Australia/VIC10/2020\|EPI_ISL_416516 | | N/A | N/A |  |
|  | Australia/VIC09/2020\|EPI_ISL_416515 | | N/A | N/A |  |
| 3-UTR (76-85) | Australia/VIC01/2020\|EPI_ISL_406844 | | N/A | N/A |  |
